# Astrocyte inhibition and PV interneuron activation: key mechanisms in electroacupuncture's effect on pain-anxiety comorbidity

**DOI:** 10.1186/s13020-025-01202-1

**Published:** 2025-09-02

**Authors:** Junfan Fang, Yashuang Xu, Mengting Qiu, Liyan Zhong, Ru Ye, Lu Guan, Junhui Ren, Zi Guo, Xiaofen He, Xiaomei Shao, Yi Liang, Jianqiao Fang, Junying Du

**Affiliations:** 1 Department of Neurobiology and Acupuncture Research, The Third School of Clinical Medicine, Zhejiang Chinese Medical University, Hangzhou, 310053 China; 2 Key Laboratory for Research of Acupuncture Treatment and Transformation of Emotional Diseases, The Third School of Clinical Medicine, Zhejiang Chinese Medical University, Hangzhou, 310053 China; 3 The First Affiliated Hospital of Zhejiang Chinese Medical University, Hangzhou, 310053 China

**Keywords:** Electroacupuncture, Comorbidity of pain-anxiety, Anterior cingulate cortex, Astrocyte-neuron interaction, Astrocyte, Parvalbumin

## Abstract

**Background:**

Evidence indicates that the interplay between pain and anxiety poses clinical challenges for the evaluation and management of chronic pain, yet effective therapies for these comorbidities are limited. This study aimed to elucidate the effects and mechanisms of electroacupuncture (EA) on pain-anxiety comorbidities.

**Methods:**

Mice injected with Complete Freund's adjuvant (CFA) in the ipsilateral hind paw developed persistent inflammatory pain and anxiety-like behaviors, as assessed by the von Frey, open field, elevated plus maze, and novelty-suppressed feeding tests. EA was administered 12-17d after CFA injection with once daily. rAAV virus and chemogenetics were used to manipulate parvalbumin (PV) interneurons and astrocytes excitation in the anterior cingulate cortex (ACC). Immunofluorescence, morphological analysis, patch clamp and in vivo fiber Ca^2+^ imaging were used to examine the activation of PV interneurons and astrocytes. The effect of EPCPX (antagonist of A1R) and chemogenetics activated astrocytes on EA analgesia were observed in a subset of mice prior to EA.

**Results:**

EA administration alleviated pain and anxiety-like behaviors in CFA mice, activated PV interneurons, and inhibited astrocytes activation in the ACC. Furthermore, both PV interneurons activation and astrocyte inhibition in the ACC elicited effects similar to those elicited by EA on pain and anxiety. Chemogenetic activation of ACC astrocytes reversed the effects of EA. Additionally, astrocyte activation in the ACC suppressed PV interneurons and induced pain-anxiety like behaviors in mice. Adenosine A1 receptors, crucial for mediating the interaction between astrocytes and PV interneurons in the ACC, were also found to be involved in the effects of EA on pain-anxiety comorbidity.

**Conclusions:**

These findings reveal that EA alleviates the pain and anxiety comorbidity through a potential mechanism involving the activation of PV interneurons, which are modulated by the inhibition of astrocytes in the ACC, thus providing a promising therapeutic strategy for persistent pain and concurrent anxiety.

**Supplementary Information:**

The online version contains supplementary material available at 10.1186/s13020-025-01202-1.

## Background

Chronic pain is a major public health concern worldwide, affecting over one third of the global population and resulting in considerable personal and socio-economic burdens [1]. Patients with chronic pain often have comorbid mental disorders such as anxiety and depression [2, 3]. Anxiety frequently coexists with chronic pain and exacerbates pain sensation [4, 5]. The interaction between pain and anxiety presents clinical challenges in the evaluation and treatment of chronic pain. Current treatments for pain-anxiety comorbidities, including opioids and antidepressants, are limited to long-term clinical use because of their adverse effects [6, 7]. Therefore, identifying effective treatment strategies for pain/anxiety comorbidities with minimal adverse effects is critical. Electroacupuncture (EA), a clinical medical technology for pain management, has fewer side effects and is clinically effective, with extensive domestic and international applications [8–10], however, the mechanism underlying pain-anxiety comorbidity remains unclear.

The anterior cingulate cortex (ACC) is a key cortical region that plays an important role in emotional responses and sensory perception. Many studies have suggested that the ACC is also involved in EA’s effect on pain-anxiety comorbidity [11–13]. EA at Zusanli (ST36) effectively suppressed sensory pain, pain-associated aversion, and anxiety-like behaviors in the C-CPA rat model by influencing NPS-NPSR protein expression in the ACC and hypothalamus [14]. EA at ST36 also produces analgesic and anxiolytic effects by modulating the rACC^Glu^-DRN pathway [15]. Parvalbumin (PV) interneurons, a critical subpopulation of γ-aminobutyric acid (GABA)-ergic interneurons in the brain, located in ACC, play an essential role in pain-anxiety comorbidity. The reduced excitability of PV interneurons in the ACC is a leading cause of chronic-inflammatory pain and anxiety [16–18]. Our previous study demonstrated that EA alleviates mechanical hypersensitivity and anxiety-like behaviors in chronic inflammatory pain by promoting the function of PV interneurons in the ACC (ACCPV) [12]. However, the mechanism by which EA upregulates the function of ACCPV to alleviate pain-anxiety comorbidities remains unclear.

Astrocytes are the most abundant type of glial cells in the central nervous system (CNS) [19]. Several studies on chronic pain models have suggested that astrocytes become overactive and implicated in the processing of the sensory and affective components of pain, including the ACC [20–22], somatosensory cortex [23], thalamus [24], and amygdala [25]. Furthermore, astrocytes play an important role in regulating synaptic function and neuronal excitability [26–28], primarily by releasing various gliotransmitters (such as ATP, D-serine, and GABA) to modulate the excitability of GABAergic interneurons [29, 30]. Notably, previous studies have suggested that the activity of PV interneurons is regulated by astrocytes [31–33] and that the response of PV interneurons is notably weakened when astrocytes are activated [34]. EA has been shown to alleviate pain or treat CNS disorders by inhibiting the activation of astrocytes in the brain [35, 36]. Therefore, the overactivation of astrocytes in the ACC may be one mechanism underlying the development of pain-anxiety comorbidities, and EA may intervene in pain-anxiety comorbidities and PV interneurons activation by modulating astrocyte activation.

Adenosine A1 receptors (A1R) are expressed postsynaptically in GABAergic interneurons [37] and inhibition of PV interneurons activity by astrocytes has been shown to be related to A1R [38]. The involvement of A1R in the mechanism of EA analgesia has been previously demonstrated [29, 30]; injecting an A1R agonist replicates the effects of EA, while injecting an A1R antagonist reverses the anti-nociceptive effects of EA [39]. Here, we hypothesized that A1R may be the critical substance linking the modulation of astrocyte by EA and PV interneuron activation in the ACC. These findings may provide important insights into the development of EA-based interventions for the treatment of pain-anxiety comorbidities.

This study aimed to investigate the underlying mechanism of EA intervention for pain-anxiety comorbidity, as it would provide theoretical evidence for clinical applications and help promote its use as a treatment modality.

## Methods

### Animals

Male adult (8–10 weeks of age) C57BL/6 mice (SLAC, Shanghai, China), PV-Cre mice (Stock No: 017320, Jackson Lab), Ai9 mice (Stock No: 007909, Jackson Lab), and PV-Cre:Ai9 mice, based on a cross between PV-Cre mice and Ai9 mice, were utilized in this study. Mice were kept in a constant ambient temperature (23 ± 2 °C, humidity: 40–60%) and ad libitum access to food and water in a 12-/12-h/dark cycle light. All experimental protocols received approval from the Animal Care and Welfare Committee of Zhejiang Chinese Medical University, Zhejiang, China (IACUC- 20200302–06).

### Stereotaxic surgery, virus injection and cannula implantation

For stereotaxic surgery, mice were deeply anaesthetized by intraperitoneal injection of 0.3% pentobarbital (80 mg/kg, Sigma-Aldrich). After fully exposing the skull suture to locate bregma and lambda, drill a hole at the target location (AP: + 1.41 mm, ML: ± 0.35 mm) under a microscope (RWD, China). Then rAAV virus (BrainTV, Wuhan, China) (Table 1) was injected into the ACC (AP: + 1.41 mm, ML: ± 0.35 mm, DV: − 0.90 mm) at a rate of 50 nL/min. The injection pipette was withdrawn from the brain 10 min after infusion. After surgery, mice recovered from anesthesia on a heating pad.

**Table 1:** Virus detail

Groups	Name	Injection location and dosage	Titer
M-PV-mCherry	rAAV-Ef1α-DIO-mCherry	Right ACC, 80 nL	5.04*1012 vg/ml
M-PV-hM3Dq	rAAV-Ef1α-DIO-hM3D(Gq)-mCherry	Right ACC, 80 nL	5.72*1012 vg/ml
C-GFAP-mCherry	rAAV-GFAP-mCherry	Bilateral ACC, 120 nL	5.94*1012 vg/ml
C-GFAP-hM3Dq	rAAV-GFAP-Cre and rAAV- Ef1α-DIO-mCherry	Bilateral ACC, 120 nL	4.69*1012 vg/ml and 5.04*1012 vg/ml
M-GFAP-mCherry	rAAV-GFAP-mCherry	Bilateral ACC, 120 nL	5.94*1012 vg/ml
M-GFAP-hM4Di	rAAV-GFAP-hM4D(Gi)-mCherry	Bilateral ACC, 120 nL	5.10*1012 vg/ml
M-GFAP-mCherry-EA	rAAV-GFAP-mCherry	Bilateral ACC, 120 nL	5.94*1012 vg/ml
M-GFAP-hM3Dq-EA	rAAV-GFAP-CRE and rAAV-Ef1α-DIO-hM3D(Gq)-mCherry 1:1	Bilateral ACC, 120 nL	5.31*1012 vg/ml, 4.69*1012 vg/ml
M-GFAP-hM4Di-PBS	rAAV-GFAP-hM4D(Gi)-mCherry	Bilateral ACC, 120 nL	5.10*1012 vg/ml
M-GFAP-hM4Di-DPCPX	rAAV-GFAP-hM4D(Gi)-mCherry	Bilateral ACC, 120 nL	5.10*1012 vg/ml

For cannula implantation, mice were stereotaxically bilaterally implanted with two 30G guide cannulas in the ACC at AP + 1.41 mm, ML ± 0.35 mm, DV − 0.90 mm. Cannulas were secured in place using dental acrylic and kept clean and unobstructed for injection using a special acupuncture needle.

For fiber photometry, AAV5-Zac2.1gfaABC1D-lck-GCaMP6f and AAV5-GFAP-hM3D(Gq)-mCherry virus were injected into the ACC at AP + 1.41 mm, ML ± 0.35 mm, DV -0.90 mm, and an optical fiber (230 μm OD, 0.37 NA) was also implanted int the ACC AP + 1.41 mm, ML ± 0.35 mm, DV -0.80 mm.

### In vivo chemogenetic manipulation

Behavioral experiments were performed at least 3 weeks after the virus injection. For neuronal inhibition, mice expressing hM4Di-mCherry or mCherry (as a control) received an intraperitoneal injection of clozapine N-oxide (CNO, 2 mg/kg; Wuhan BrainTVA, China) 30 min before the behavioral tests. For neuronal activation, hM3D (Gq)-mCherry or mCherry (control) mice received an intraperitoneal injection of CNO 30 min before behavioral tests.

### Cannula infusion experiment

Four weeks after virus injection and cannula implantation surgery, DPCPX or artificial cerebrospinal fluid (ACSF) was microinjected into the ACC for consecutive 7 days starting at 12 days after Complete Freund’s adjuvant (CFA) injection. For intracranial injections [40], the internal cannulas were connected to a 10 µL microsyringe mounted on a microinfusion pump. The infusion rate was 200 nL/min for an infusion volume of 1 μL. The microsyringe remained in situ for an additional 3 min to allow drug diffusion after microinjection and was then slowly removed. Only dara from mice with correctly positioned injections was used.

### Drug administration

DPCPX (Sigma-Aldrich) was suspended in PBS at a dose of 0.3 mg/kg for systemic injection. CCPA (Sigma-Aldrich) was suspended in PBS at a dose of 0.1 mg/kg for systemic injection. The volumes of DPCPX and CCPA used for systemic injection were 0.2 mL. DPCPX (Sigma-Aldrich) was suspended in ACSF (NaCl 126 mM, KCl 2.5 mM, NaHP2O4 1.2 mM, NaHCO3 26 mM, MgCl2 1.25 mM, CaCl2 2 mM, and glucose 10 mM) for final concentrations of 0.03 ug/μL for intra-ACC infusions. The volume of DPCPX used for intra-ACC infusion was 1 μL. Clozapine N-oxide (CNO, 0.2 mg/ml, 0.2 mL) was injected intraperitoneally at a dose of 2 mg/kg.

### Establishment of a chronic inflammatory pain model

CFA (20 µL, at 4 mg/mL, 1:1 PBS, Chondrex, USA) was injected into the left subcutaneous plantar to establish the chronic inflammatory pain model. The same amount of PBS was injected in the same manner as in control mice.

### Establishing a neuropathological pain model

Mice were anesthetized using 2% isoflurane via inhalation. The left hind limb was shaved, and the skin was disinfected using iodophor. A precise incision was made midway between the tibial head and the greater trochanter of the femur to expose the sciatic nerve through blunt dissection using precision forceps. The sural and common peroneal nerves were trimmed to 2–3 mm, whereas the tibial nerve remained intact. In the Sham group, the nerves were left uncut, following the same procedure as in the model group. The incision was sutured and treated using iodophor. Subsequently, the mice were placed on a 37 °C thermostatic pad to monitor their vital signs and allow for recovery from anesthesia.

### EA interventions

The EA intervention was conducted from day 12 until the end of the experiment. The mice were fixed and disinfected at a radius of 1 cm and centered at the acupoints. Acupuncture needles (specification 0.16 × 7 mm) were inserted at bilateral Zusanli (ST36, located around 4 mm inferior from the knee joint, 2 mm lateral to the anterior tubercle of the tibia) and nearby position (ST 36 below 0.5 cm) to a depth of 5 mm, and then the ipsilateral acupoint was connected by HANS-200A Acupoint Nerve Stimulator (Huawei Co, Ltd, China). The EA stimulation parameters were as follows: 2/100 Hz, 0.2–0.4 mA (stimulus intensity was initially 0.2 mA and increased by 0.1 mA every 10 min of treatment).

Mice in the sham EA group were administered acupuncture needles at the subcutaneous acupoints without applying any current. In the combined chemogenetic EA experiments, CNO was injected intraperitoneally followed by EA intervention. All mice were prepared using the same fixation method.

### Paw withdrawal thresholds

Paw withdrawal thresholds (PWTs) were assessed using the “up-down” method [41]. The animals were placed in transparent boxes on an elevated mesh floor for at least 30 min to adapt to the environment. Subsequently, an increasing series of Von Frey hairs (0.02, 0.04, 0.07, 0.16, 0.4, 0.6, 1.0, 1.4, and 2.0 g) was used during the test. The first hair used was 0.4 g. The midplantar surface of the hind paw was perpendicularly stimulated for 6–8 s in the experiment, and abrupt withdrawal or licking or shaking of the foot was considered a positive response and recorded as “X,” while negative responses were recorded as “O.” Four more stimuli were used after the first “OX” or “XO” occurred. The results were calculated using the following formula: PWTs (g) = [10(Xf + κδ)]/10,000. “xf” is the logarithmic value of the force of the last test hair, the “κ” value is obtained from the κ-value table, and “δ” is the mean difference between the logarithm of hairs of each force, here is 0.244.

### Open field test

The mice were placed in the center zone of the open field test (OF) chambers (40 × 40 × 40 cm), and their behavior was recorded for 5 min using a camera. The light intensity was set to 200 lx. ANY-Maze software (version 6.0; Stoelting, USA) was used to analyze the videos. The duration of time spent in the central zone, distance traveled within the central zone, and number of entries into the central zone were analyzed to assess the anxiety levels of the animals. The total distance was analyzed to assess locomotion. Between each test, the apparatus was cleaned with 10% ethanol to remove olfactory information. The anxiety index was calculated [42] as follows: Anxiety index = $$1-(\left(time in central zone\div total time\right) +\left(number of entries into the central zone\div total entries\right))\div 2$$.

### Elevated plus maze (EPM)

The maze was constructed of light-gray polyethylene and was elevated 50 cm above the floor. It consisted of a square center region (6 × 6 cm) connected by two opposing closed arms (30 × 6 × 10 cm) and two open arms. The light intensity was set to 200 lx. The mice were gently placed in the center platform of the maze, and a video camera was recorded for 5 min. The duration spent in the open arm and the number of entries into the open arm were calculated using the ANY-Maze software (version 6.0; Stoelting, USA). Between each test, the apparatus was cleaned with 10% ethanol to remove olfactory information. The anxiety index was calculated [42] as follows: Anxiety index = $$1-(\left(time in open arms \div total time\right) + \left(number of open arms entries \div total entries\right))\div 2$$

### Emotionality z score

The z scores were calculated using the following formula [43]: z = (X − μ)/σ. X represents the individual data for the observed parameter, where μ and σ represent the mean and the standard deviation for the control group, respectively. Behavioral parameters related to emotional aspects (time spent in the center zone in the OF and time spent in the open arms in the EPM) were used to calculate the z score for each animal in each test. The emotionality z score for each animal was calculated as the average of the z scores from the OF and EPM tests.

### Novelty-suppressed feeding test (NSF)

After 24 h of food deprivation, mice were habituated for 1 h in the testing room. An open-topped black box (40 × 40 × 30 cm) with a light intensity of 1000–1050 lx was used. After putting a food pellet crushed into 1.0–2.0 g at the center of the arena, the mice were placed at the corner of the arena. Latency to feed was measured as the amount of time elapsed before biting the food pellet. If the mice did not begin feeding within 5 min, feeding was recorded at 300 s. After the NSF test, the mice were placed in a cage with a pre-weighed food pellet. After 30 min, the food pellets were reweighed to determine the home cage consumption. Between each test, the apparatus was cleaned with 10% ethanol to remove olfactory information.

### Fiberphotometry recording and analysis

A fiber photometry system (Thinker Tech Nanjing Bioscience Inc.) was used for data acquisition. The animals were acclimated to the recording environment for an hour of habituation before undergoing a baseline assessment for 30 min. Subsequently, the mice were administered saline or CNO injections and the results were recorded for 45 min. Following the recordings, the raw data were exported and processed by applying a 100 Hz moving average filter for smoothing. Delta F/F was then calculated at 30 min intervals before and after administration of saline or CNO [44]. Peaks that surpassed the threshold (set at 3 MADs above the median) were detected, quantified, summed, and normalized by recording their duration to determine the peak frequency [45].

### Immunohistochemistry

After the last behavioral measurement (CNO intervention within 2 h), the mice were immediately anesthetized using pentobarbital (80 mg/kg, i.p.) and transcardially perfused with phosphate buffered saline (PBS), followed by 4% paraformaldehyde (PFA) in PBS. Brains were extracted and postfixed overnight in 4% PFA at 4 °C. Fixed brains were cryoprotected with 30% sucrose in PBS for 2 days at 4 °C and then sectioned on a cryostat (Thermo, NX50) into 25 μm coronal slices. The sections were washed in PBS six times for 10 min each, incubated in a blocking solution (10% normal donkey serum in PBS containing 0.3% Triton X-100) for 1 h at 37 °C, and incubated with primary antibodies in the blocking solution for 18 h at 4 °C. The following primary antibodies were used: rabbit anti-c-Fos (1:1000, ab190289, Abcam, USA), rabbit anti-PV (1:500, ab181086, Abcam, USA), rabbit anti-GFAP (1:1000, ab7260, Abcam, USA), rabbit anti-Iba1 (1:1000, ab178864, Abcam, USA), rabbit anti-NeuN (1:1000, ab177487, Abcam, USA), and rabbit anti-adenosine A1R (1:200, NB300549, Novus Biologicals, USA). After washing six times in PBS for 10 min each, the sections were incubated with donkey anti-rabbit IgG H&L (Alexa Fluor® 488) (1:800, ab150061, Abcam, USA) in the blocking solution for 1 h at 37 °C. After washing with PBS six times for 10 min each, the slides were covered with a mounting medium containing 4′,6-diamidino-2-phenylindole (DAPI; H-1500; Vector Laboratories, Burlingame, CA, USA). Fluorescence images were acquired using an Imager M2 microscope (ZEISS). Cell counting was conducted by a researcher who was blinded to the group allocation, utilizing the Cell Counter plugin in the ImageJ software (National Institutes of Health, Bethesda, MD, USA).

### Image processing and analysis

For statistical analysis, 3–5 mice (5–6 images per mouse), were randomly chosen from each group by an experimenter blinded to the group allocation. The expression of c-Fos, PV, Iba1 positive cells, GFAP positive area, morphology analysis, and Sholl analysis was performed using ImageJ software [46] (National Institutes of Health, Bethesda, MD, USA). For morphological and Sholl analysis of GFAP, 10 individual cells were randomly selected from each mice [47]. Sholl analysis was performed as described by Ferreira et al. [48]. The color of the selected original images was converted to 8-bit, the threshold was adjusted, and background noise was eliminated. The images were then binarized, and the cell skeleton was depicted. Finally, a series of concentric circles was drawn with a step length of 1 μm from the center of the cell, and the number of intersections between each concentric circle and the cell skeleton was recorded.

### Western blotting

After the final behavioral assessment (conducted within 2 h of the CNO intervention), the mice were promptly anesthetized with pentobarbital (80 mg/kg, i.p.) and transcardially perfused with phosphate-buffered saline (PBS). The brains were excised, and the ACC was isolated. Tissue samples were homogenized in RIPA buffer, followed by centrifugation at 14,000 rpm for 5 min at 4 °C. The protein concentrations were determined using the BCA method. Lysates containing 15 µg of protein were denatured at 95 °C for 5 min, electrophoresed on a 10% SDS-PAGE gel, and then transferred onto polyvinylidene difluoride (PVDF) membranes (Merck KGaA, Darmstadt, Germany). The membranes were subsequently blocked with 5% low-fat milk in TBST for 1 h at room temperature, followed by overnight incubation at 4 °C with rabbit anti-Adenosine A1R antibody (1:500, NB300549, Novus Biologicals), rabbit anti-A2AR (1:500, sc-32261, Santa Cruz), or mouse anti-β-actin (1:5000, YM3028, Immunoway). Subsequently, the membranes were treated with peroxidase AffiniPure™ goat anti-rabbit or mouse IgG (H + L) for 2 h at room temperature. An ECL kit (Pierce, Rockford, IL, USA) was utilized for visualization. Band quantification was performed using FIJI; the results were presented as mean ± SEM.

### Electrophysiological recording

The experimental techniques were similar to those previously reported [49]. Mice injected with the hybrid virus of rAAV-GFAPARCID-hM3D(Gq)-mCherry and rAAV-PV-EGFP were anesthetized using isoflurane and decapitated. Coronal brain slices (300 μm thickness) were prepared using a Leica VT-1200S vibrated slicer in an ice-cold cutting solution containing the following (in mM): 2.5 KCl, 1.25 NaH_2_PO_4_, 7 MgCl_2_, 25 NaHCO_3_, 25 glucose, 0.5 CaCl_2_, 75 sucrose, equilibrated using 95% O_2_–5% CO_2_. Acute slices were maintained in artificial cerebrospinal fluid (ACSF) for 30 min at 34 °C and then brought to room temperature until they were transferred to a recording chamber. Artificial cerebrospinal fluid (ACSF) for slice maintenance (in mM): 126 NaCl, 26 NaHCO_3_, 10 D-glucose, 2.5 KCl, 2.5 CaCl_2_, 1.25 MgCl_2_, 1.25 NaH_2_PO_4_. For loose-seal recordings, a modified ACSF was used (in mM): 130 NaCl, 3.5 KCl, 0.5 MgCl_2_, 1.25 NaH_2_PO_4_, 26 NaHCO_3_, 2.0 CaCl_2_, 10 D-glucose. Typically, three brain slices containing the ACC with a fluorescence signal were obtained from each animal, and recordings were made at different levels throughout this brain region. Slices were transferred into a chamber maintained at 32–35 °C (TC-324B, Warner), and the PV interneurons were identified using a Nikon microscope (N1R, Japan) by fluorescence emission and then visually targeted with infrared gradient contrast optics. Loose cell-attached recordings (seal resistance: 20–70 MΩ) were made using 4–6 MΩ borosilicate glass pipettes containing normal ACSF in the presence of excitatory transmission blockers (10 μM bicuculline and 50 μM D-APV) at a holding potential of 0 mV. Signals were filtered at 6 kHz and acquired using pClamp software (Molecular Devices) in the voltage clamp mode at a sampling rate of 10 kHz. In most cases, the recordings were initiated 5 min after establishing loosely sealed cell-attached recordings. The firing rate during the last minute before introducing CNO perfusion was used to calculate the pre-drug application firing rate. CNO was administered via perfusion for at least 5 min, and the firing rate during the last minute before washing was used to calculate the firing rate during drug application. Following a 3-min wash with ACSF, the firing rate of the PV interneurons was calculated as the post-CNO application rate.

### Statistical analysis

The data were presented as mean ± SEM. Statistical analyses were performed using SPSS version 20.0. We conducted a two-tailed Student’s t-test for experiments with only two groups, one-way analysis of variance (ANOVA) with post-hoc Tukey’s multiple comparison test for single-factor experiments with > two groups, and two-way ANOVA followed by multiple comparisons with post-hoc Tukey’s test or Bonferroni’s test for double-factor experiments. A two-way repeated-measures ANOVA with post-hoc Tukey’s multiple-comparison test was used for data in the PWTs conducted at multiple time points. *P* < 0.05 was considered statistically significant.

## Results

### EA at ST36 attenuates pain and anxiety-like behaviors of CFA mice

**Fig. 1:**
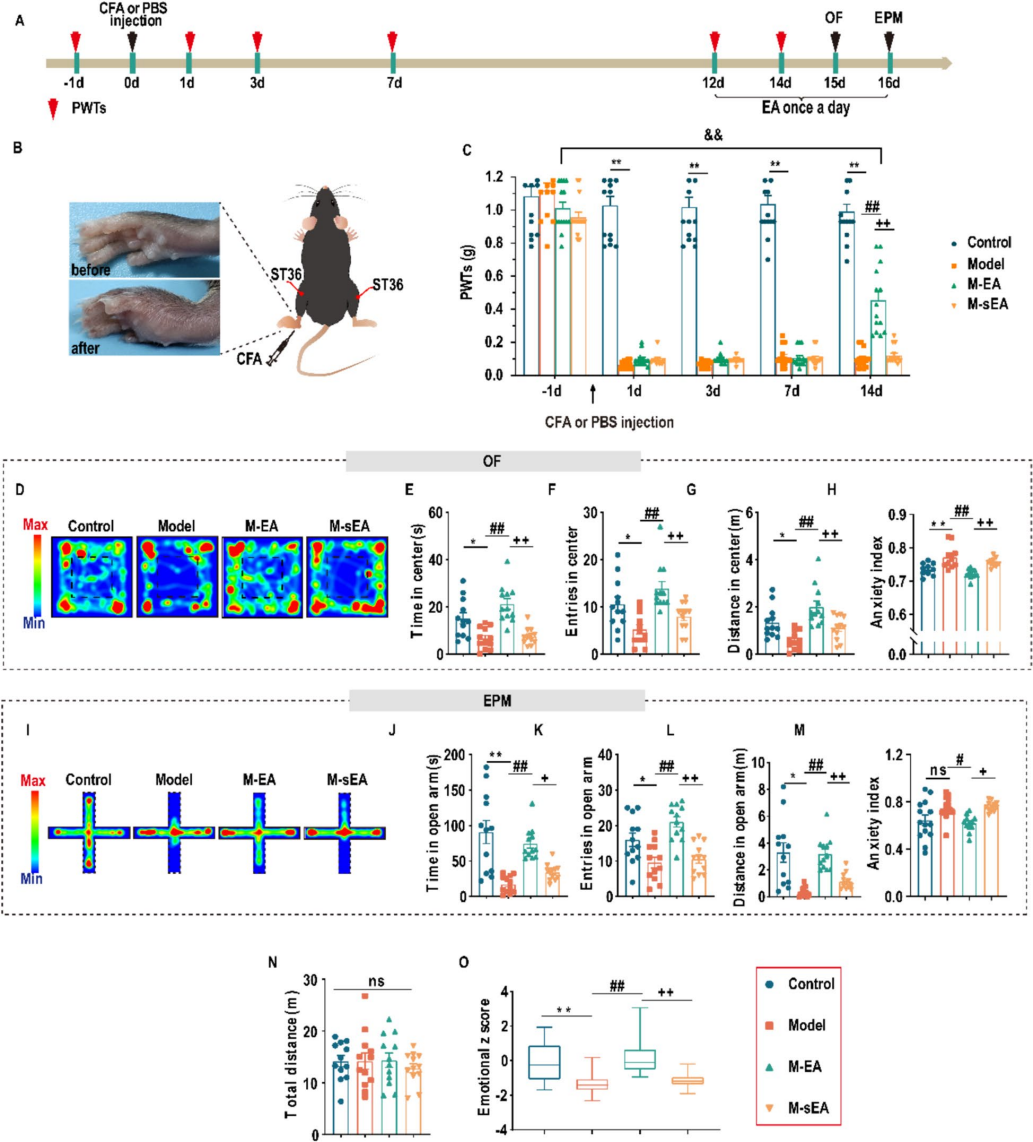
EA attenuates pain and anxiety-like behaviors of CFA mice. **A** Schedule for the procedure and subsequent experiments. **B** Schematic of the CFA model. **C** The effect of EA on PWTs (F_3,52_ = 214.2, two-way ANOVA with Tukey’s multiple comparisons test, *P* < 0.0001; n = 14 mice/group). **D**–**H** Result of OF. D Representative locomotion trace in OF. **E** Time in center of OF (F_3,44_ = 14.25, *P* < 0.0001). **F** Entries in center of OF (F_3,44_ = 9.059, *P* < 0.0001). **G** Distance in center of OF (F_3,44_ = 10.72, *P* < 0.0001), **H** Anxiety index of OF (F_3,41_ = 12.27, *P* < 0.0001). **I**–**M** The result of EPM. **I** Representative exploration traces in EPM. **J** Time in open arm of EPM (F_3,44_ = 14.26, *P* < 0.0001). **K** Entries in open arm of EPM (F_3,44_ = 11.77, *P* < 0.0001). **L** Distance in open arm of EPM (F_3,44_ = 12.37, *P* < 0.0001). **M** Anxiety index of EPM (F_3,48_ = 5.960, *P* = 0.0015). **N** Effect of EA on total distance in the OF (F_3,44_ = 0.3266, *P* = 0.8061). **O** Effect of EA on emotional z score (F_3,92_ = 23.42, *P* < 0.0001). *P < 0.05, **P < 0.01, Control v.s. Model; #* P* < 0.05, ##* P* < 0.01, Model v.s. M-EA; + *P* < 0.05, +  + *P* < 0.01, M-EA v.s. M-sEA. ns, no significant different (*P* > 0.05). Data are presented as the means ± SEM. n = 12–14 mice/group. One-way ANOVA (**E**–**H**, **J**–**O**) with Tukey’s multiple comparisons test

The initial focus of our study was to investigate the effects of EA on pain in CFA-induced mice using the von Frey test. The von Frey test was performed at -1 day and 1 day after CFA paw injection to validate pain induction (Fig. 1A). Over 14 days, the CFA-injected mice exhibited a decreased mechanical stimulus threshold in the ipsilateral hind paw. EA intervention led to an increase in PWTs, whereas sham EA intervention had no effect on the PWTs (Fig. 1A–C). However, after EA intervention, the PWTs of mice with inflammation remained lower than those prior to injection of CFA (Fig. 1C).

After a 14-day period following CFA injection, anxiety-like behavior was assessed using OF and EPM tests to evaluate the impact of EA (Fig. 1A). In the OF test, CFA-injected mice exhibited fewer entries and spent less time in the center area; however, EA intervention led to an increase in both the number of entries and time spent in the center area (Fig. 1D–H). Additionally, CFA-injected mice displayed an anxiety-like phenotype in the EPM test, characterized by a reduced duration and number of entries in the open arm. EA intervention resulted in an increase in the time spent in the open arm and the number of entries into the open arm (Fig. 1I–M). There were no significant differences in locomotor activity, as measured by the total distance traversed in the OF test, among the four groups (Fig. 1N). Moreover, we calculated emotionality z scores to evaluate the consistency of the behaviors observed in these tests. The emotionality z score was significantly lower in the Model group than that in the Control group. The EA intervention resulted in an increased emotionality z score, whereas the sham EA intervention had no effect (Fig. 1O). Concurrently, the impact of EA on pain hypersensitivity and anxiety-related behaviors in the OF and EPM tests was evaluated in mice with neuropathic pain induced by spared nerve injury (SNI). As anticipated, EA treatment significantly ameliorated pain hypersensitivity and anxiety-like behaviors, whereas sham EA treatment had no effect (Figure S1). Collectively, these findings demonstrated that EA significantly alleviates pain and reduces anxiety.

We further examined whether the efficacy of EA depended on stimulus intensity. Both the 0.5 mA and 3 mA EA interventions provided effective analgesia and alleviated negative emotions (Figure S2); however, their effects did not differ significantly.

### Functional role of the ACCPV in analgesia and relieving emotion by EA

Our previous study demonstrated that ACCPV play a role in the effects of EA on pain and pain-related anxiety-like behavior [12]. In this study, we examined c-Fos positive and PV positive cells in the ACC. The number of c-Fos positive cells increased in the bilateral ACC of CFA-injected mice compared to Control mice (Figure S3A–B), whereas the number of PV-positive cells decreased in the bilateral ACC of CFA-injected mice compared to Control mice (Figure S3C–D). EA intervention resulted in a decrease in the number of c-Fos positive cells and an increase in the number of PV positive cells in the bilateral ACC, consistent with previous studies [51, 52] (Figure S3).

**Fig. 2:**
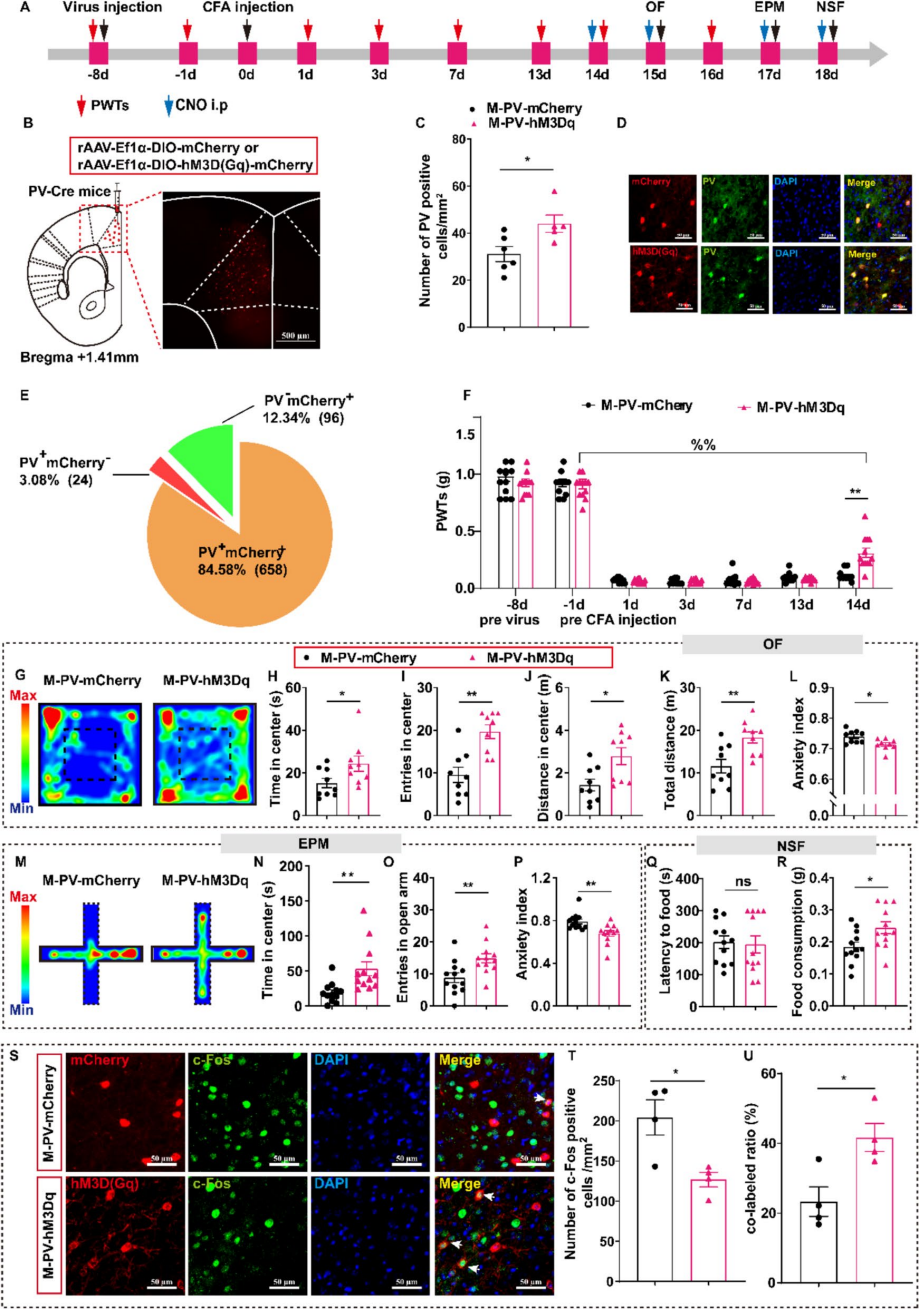
Chemogenetic activating of PV interneurons directly alleviates the pain-anxiety comorbidity and inhibits the c-Fos expression. **A** Experimental scheme of virus injection, CFA modeling, and behavioral tests. **B** Virus injection strategy (left) and representative images show locations of virus expression (right). **C** The number of PV positive cells expression (t = 2.672, P = 0.0256), **D** Representative image of co-labeling for mCherry and PV in the ACC, allow showed mCherry + , PV interneurons. Bar = 50 μm. **E** Quantitative analysis of co-labeling of mCherry and PV in the ACC. **F** Result of PWTs (on the 14d, t = 4.602, P = 0.0001). **G**–**L** Results of OF. G Representative exploration traces of OF. **H** Time in center of OF (t = 2.180, P = 0.0446). **I** Entries in center of OF (t = 4.205, P = 0.0007). **J** Distance in center of OF (t = 2.795, P = 0.0130). **K** Total distance of OF (t = 3.210, P = 0.0055). **L** Anxiety index of OF (t = 2.814, P = 0.0125). **M**–**P** Results of EPM. M Representative exploration traces in EPM. **N** Time in open arm of EPM (t = 3.155, P = 0.0046). **O** Entries in open arm of EPM (t = 2.925, P = 0.0078). **P** Anxiety index of EPM (t = 3.135, P = 0.0048). **Q**, **R** Results of NSF. **Q** Latency to food (t = 0.2138, P = 0.8326). **R** Food consumption (t = 2.546, P = 0.0184). ^%%^*P* < 0.01, compared with the pre-CFA injection of M-PV-hM3Dq; *P < 0.05, **P < 0.01, M-PV-mCherry v.s. M-PV-hM3Dq; ns, no significant different (*P* > 0.05). Data are presented as the means ± SEM. n = 9–12 mice/group. Two-tailed unpaired *t*-test. **S** Representative images of c-Fos positive cells in ACC. **T** Quantitative analysis of c-Fos positive cells in ACC (t = 3.261, P = 0.0172); **U** Ratio of co-localization between mCherry and c-Fos (t = 3.158, P = 0.0196). n = 4 mice/group. Two-tailed unpaired *t*-test. *P < 0.05, M-PV-mCherry v.s. M-PV-hM3Dq

We further investigated whether the activation of ACCPV could simulate the effects of EA on pain and pain-related anxiety in mice. We unilaterally injected rAAV-Ef1α-DIO-hM3D (Gq)-mCherry into the ACC of PV-Cre mice and assessed the pain and anxiety-like behavior following activation of ACCPV through administration of CNO (Fig. 2A, B). Immunofluorescence examination verified that the number of PV positive cells increased in the ACC of the M-PV-hM3Dq group compared to that in the M-PV-mCherry group, and mCherry (virus) was primarily restricted to PV interneurons in the ACC (Fig. 2C–E). We also found that chemogenetic activation of ACCPV increased the nociceptive threshold to mechanical stimulation in CFA-injected mice but remained lower than that prior to CFA injection (Fig. 2F). ACCPV activated mice also exhibited fewer anxiety-like behaviors in the OF, EPM, and NST tests and increased locomotor activity in the OF test, but did not alter the latency to food consumption (Fig. 2G–R). Additionally, the M-PV-hM3Dq group exhibited a decrease in the number of c-Fos positive cells in the ACC (Fig. 2S–T). Moreover, there was an increased ratio of co-localization between mCherry and c-Fos in the M-PV-hM3Dq group compared to that in the M-PV-mCherry group (Fig. 2U). Overall, these results showed that the activation of ACCPV resulted in analgesic effects, relief from anxiety, and a reduction in c-Fos-positive cells. These effects suggest that EA may modulate the comorbidity of pain and anxiety by activating ACCPV, resembling the effects of EA stimulation on pain-anxiety comorbidity [12].

Therefore, we chemogenetically inhibited ACCPV to block the analgesic and anxiolytic effects of EA in CFA injected mice. By inhibiting ACCPV, EA treatment did not alleviate pain and anxiety-like behaviors in CFA-injected mice (Figure S4). Collectively, these results indicate that the inhibition of ACCPV plays a crucial role in the regulatory influence of EA on pain-anxiety comorbidity in a CFA-induced mouse model.

### Astrocytes in the ACC play an important role in processing pain-anxiety comorbidity

**Fig. 3:**
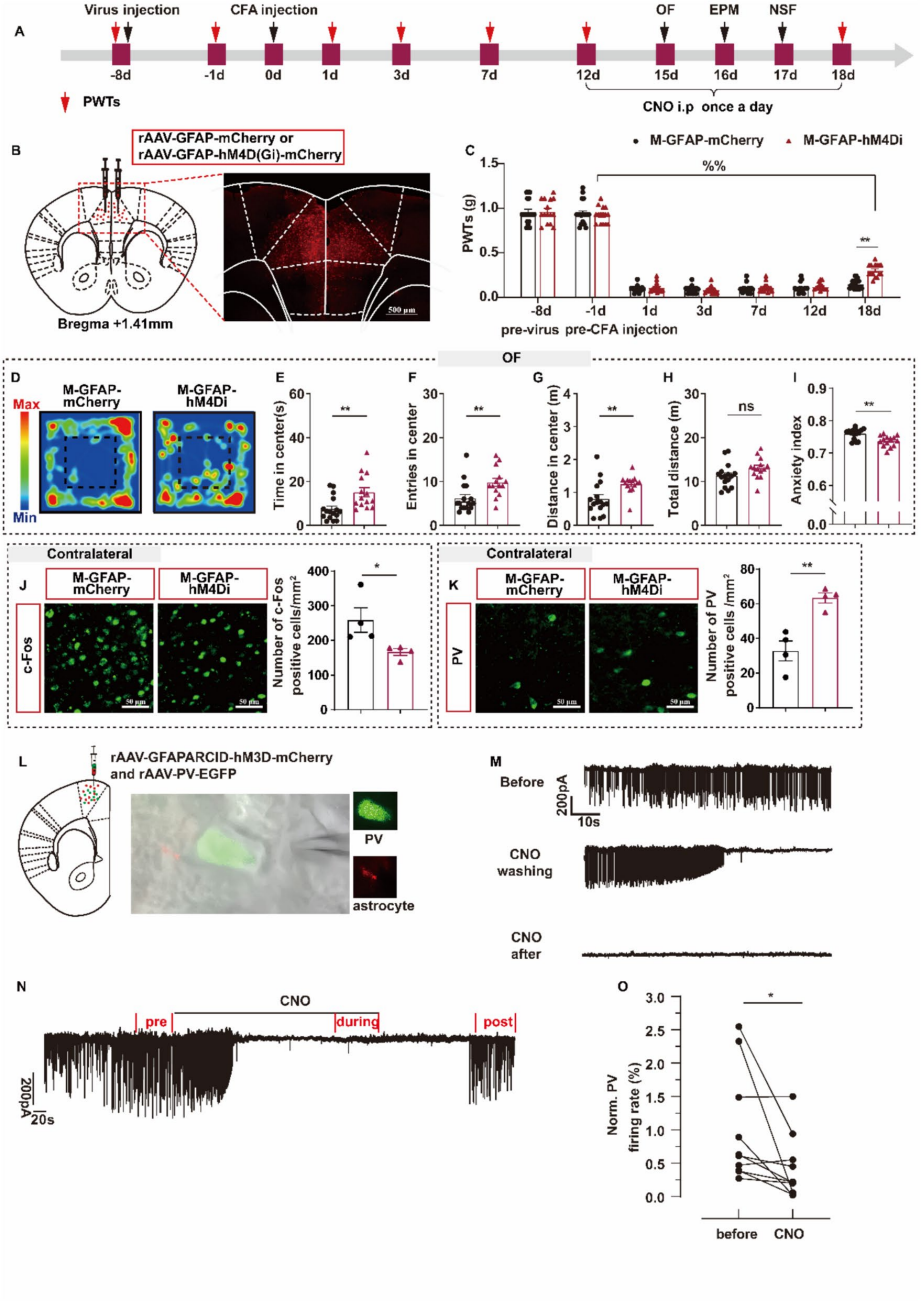
Chemogenetic inhibiting astrocyte activation in the ACC relieve pain-anxiety comorbidity and increase the ACCPV. **A** Experimental scheme of virus injection, CFA modeling, and behavioral tests. **B** Virus injection strategy (left) and representative images show locations of virus expression (right). **C** Inhibition of astrocytes decreased the PWTs (on 18d, t = 6.867, P < 0.0001). **D**–**I** Results of OF. D Representative exploration traces of OF. E Time in center of OF (t = 3.108, P = 0.0043). **F** Entries in center of OF (t = 2.878, P = 0.0076). **G** Distance in center of OF (t = 2.950, P = 0.0064). **H** Total distance (t = 1.878, P = 0.0709). **I** Anxiety index of OF (t = 3.708, P = 0.0009). n = 14–19 mice/group. Two-tailed unpaired *t*-test. **J** Inhibition of astrocytes activation decreased the c-Fos positive cells in contralateral ACC (t = 2.500, P = 0.0465). **K** Inhibition of astrocytes activation decreased PV positive cells in contralateral ACC (t = 4.753, P = 0.0031). n = 4 mice/group. ^%%^*P* < 0.01, compared with the pre-CFA injection of M-GFAP-hM4Di; *P < 0.05, **P < 0.01, M-GFAP-mCherry v.s. M-GFAP-hM4Di; ns, no significant different (*P* > 0.05). Data are presented as the means ± SEM. **L** Virus injection strategy (left) and representative images show patch cell. **M**–**N** a sample traces shows that addition of CNO (10 μM) to the acute brain slices from the mice infused rAAV-GFAPARCID-hM3D-mcherry and rAAV-PV-EGFP significantly reduced the firing rate of PV interneuron. **O**. statistical result of the effect of astrocyte activation on firing rate of PV interneuron (t = 2.364, P = 0.0423), 9 neurons from 3 mice. *P < 0.05, before v.s. CNO

Previous research has suggested that, in addition to neurons in the ACC involved in pain-anxiety comorbidity, glial cells, particularly astrocytes, may also play a role in this comorbidity [50]. Therefore, we first investigated whether astrocyte inactivation indeed relieves pain-anxiety comorbidities and increases PV expression, and bilaterally infected C57BL mice with AAV-GFAP-hM4D(Gi)-mCherry, which specifically inhibited astrocyte activation (Fig. 3A–B). IF examination verified that hM4Di-mCherry expression was primarily restricted to astrocytes (91.13%) but not to neurons and microglia in the ACC (Figure S5). No significant differences in PWTs were observed between the M-GFAP-mCherry and M-GFAP-hM4Di groups at any time point prior to CNO administration (Fig. 3C). Inactivation of astrocytes by CNO administration significantly increased the PWTs of mice on day 18 after CFA injection compared with those of the control virus-injected group, but the PWTs were lower than those before CFA injection (Fig. 3C). In addition, inhibition of astrocytic activity promoted resilience to anxiety-like behavior in the OFT, EPM, and NSF (Fig. 3D–I, Figure S6A–E). There were no significant differences in the total distance traveled in the OF or food intake in the NSF between the M-GFAP-mCherry and M-GFAP-hM4Di groups (Fig. 3H, Figure S6F). Moreover, the inhibition of astrocyte activity resulted in an increase in emotional z scores (Figure S6G). These results suggest that astrocyte deactivation alleviates pain and reduces anxiety-like behavior induced by CFA injections.

Additionally, we observed that the inhibition of astrocytice activity affected ACC neuron activity and PV expression. The results demonstrated a decrease in the number of c-Fos positive cells and an increase in the number of PV positive cells in the M-GFAP-hM4Di group (Fig. 3J–K, Figure S6H–I). A loose-seal patch combined with a chemical-genetic DREADD approach was used to further investigate the relationship between PV interneurons and astrocytes (Fig. 3L). Using an in vitro protocol, we performed loose-patch clamp recordings of PV interneurons in acute brain slices from GFAP-hM3Dq-mCherry and PV-EGFP mice (Fig. 3L). We observed that the exposure of ACC brain slices from GFAP-hM3Dq mice to CNO (10 mM) led to a prominent reduction in the action potential firing rate of PV interneurons, which lasted several minutes (Fig. 3M–O). Collectively, these findings indicate the involvement of astrocytes in the ACC in pain-anxiety comorbidity and the potential of inhibiting astrocyte activation to induce PV-positive cell excitation, which is like the effects of EA in pain-anxiety comorbidity.

**Fig. 4:**
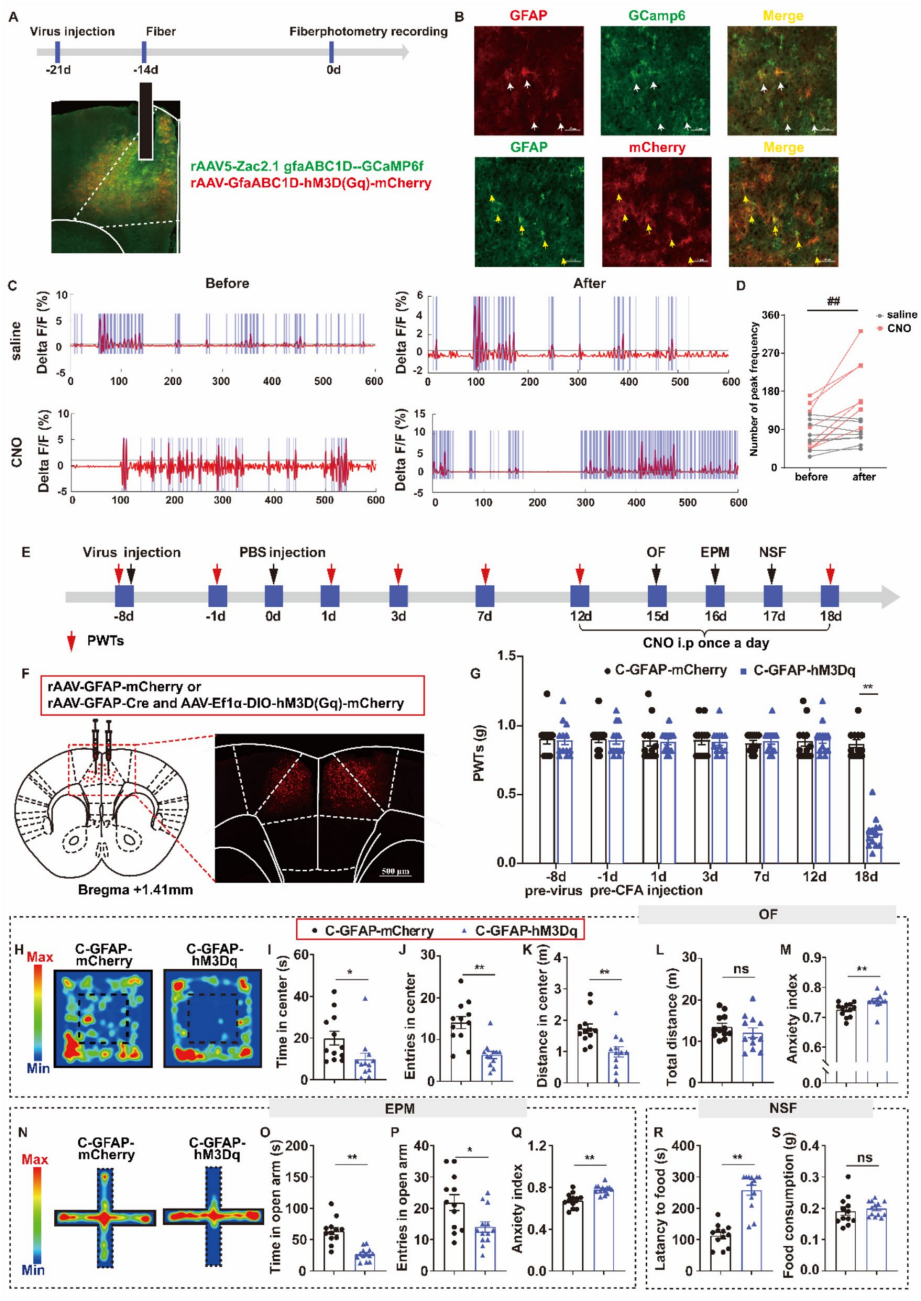
Activation of astrocytes in the ACC can induce pain sensation and pain-related anxiety-like behaviors. **A** Experimental scheme of virus injection (up) and representative images show locations of virus expression (down). **B** The representation of virus specific expression. White arrows indicate infected cells express GCaMP (green) and co-labeled with GFAP + cells (red). Yellow arrows indicate infected cells express mCherry (red) and are co-labeled with GFAP + cells (green) bar = 50 μm. **C** Representative 600 s trace recording of calcium transients before and after acute saline or CNO administration. Brue line indicates local maxima peaks that are 3 median calcium transients (MAD) above the median. **D** Data represents peak frequency for individual recording for each animal before and after injection saline or CNO (saline t = 0.3852, P = 0.7115; CNO t = 6.105, P = 0.0005). n = 8 mice/group, ## before v.s. after. **E** Experimental scheme of virus injection, PBS injection, and behavioral tests. **F** Virus injection strategy (left) and representative image show location of virus expression (right). **G** Activation of astrocytes inhibited the PWTs (on 18 d, t = 15.05, P < 0.0001). **H**–**M** Results of OF. **H** Representative exploration traces of OF. **I** Time in center of OF (t = 4.643, P = 0.0001). **J** Entries in center of OF (t = 3.108, P = 0.0053). **K** Distance in center of OF (t = 3.687, P = 0.0014). **L** Total distance of OF (t = 11.096, P = 0.2857). **M** Anxiety index of OF (t = 4.536, P = 0.0002). N-S Result of EPM. **N** Representative exploration traces in EPM. **O** Time in open arm of EPM (t = 3.748, P = 0.0012). **P** Entries in open arm of EPM (t = 2.672, P = 0.0143). **Q** Anxiety index of EPM (t = 3.586, P = 0.0017). **R**–**S** Result of NSF. **R** Latency to food (t = 3.850, P = 0.0009). **S** Food consumption (t = 0.4108, P = 0.6854). *P < 0.05, **P < 0.01, C-GFAP-mCherry v.s. C-GFAP-hM3Dq; ns, no significant different (*P* > 0.05). Data are presented as the means ± SEM. n = 12–13 mice/group. Two-tailed unpaired *t*-test

Next, we investigated whether the activation of astrocytes in the ACC alone could induce pain sensation and pain-related anxiety-like behaviors. To validate astrocytes (GFAP^+^ cell) activation in the ACC, we first conducted calcium imaging in animals infused with rAAV5-Zac2.1gfaABC1D-GCaMP6f, and rAAV5-GFAP-hM3D(Gq)-mCherry to identify changes in GFAP + calcium transients before and after the administration of saline or CNO (Fig. 4A). We found an increase in the peak frequency of GFAP^+^ calcium activity after CNO injection (P = 0.0005), but not after saline injection (P = 0.7115). The expression and cell specificity of rAAV5-Zac2.1 gfaABC1D-GCaMP6f, and rAAV5-GFAP-hM3D(Gq)-mCherry were confirmed (Fig. 4B–D). We also used this approach to test whether enhanced astrocytic activity induced pain sensitization and anxiety-like behaviors (Fig. 4E–F). We observed a decrease in PWTs and an increase in anxiety-like behavior in the OF, EPM, and NSF of mice in the C-GFAP-hM3Dq group after administration of CNO compared to the C-GFAP-mCherry group (Fig. 4G–S). However, the total distance traveled did not differ between the C-GFAP-mCherry and C-GFAP-hM3Dq groups (Fig. 4L). These findings suggest that the activation of astrocytes in the ACC can induce pain sensation and pain-related anxiety-like behaviors. All the above results established a connection between astrocyte activation in the ACC and the comorbidity of pain and anxiety.

**Fig. 5:**
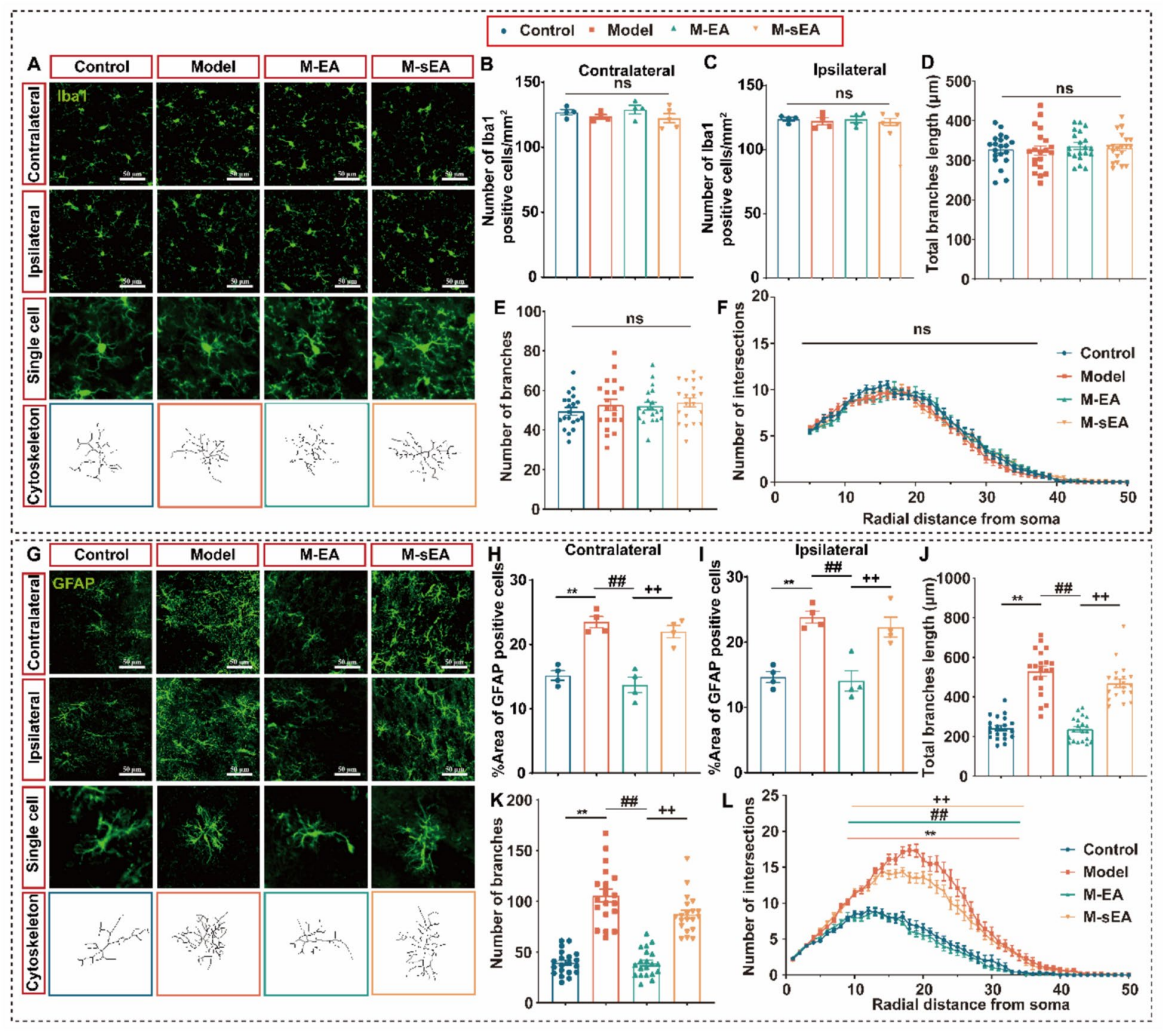
EA treatment inhibits astrocytes activation. **A** Respective image of Iba1 in the ACC. **B** Iba1 expression levels in contralateral ACC (F_3,13_ = 1.151, P = 0.3654). **C** Iba1 expression levels in ipsilateral ACC (F_3,13_ = 0.1908, P = 0.9008). **D** Total branches length of Iba1 in ACC (F_3,76_ = 0.2740, P = 0.8440). **E** Number of branches of Iba1 in ACC (F_3,76_ = 0.7056, P = 0.5516). **F** Number of intersections of Iba1 in ACC. **G** Respective images of GFAP in the ACC. **H** % Area of GFAP positive cells in contralateral ACC (F_3,12_ = 25.97, P < 0.0001). **I** % Area of GFAP positive cells in ipsilateral ACC (F_3,12_ = 16.59, P < 0.0001). **J** Total branches length of GFAP in ACC (F_3,76_ = 64.99, P < 0.0001). **K** Number of branches of GFAP in ACC (F_3,76_ = 63.05, P < 0.0001). **L** Number of intersections of GFAP in ACC. **P < 0.01, Control v.s. Model; ##*P* < 0.01, Model v.s. M-EA; +  + *P* < 0.01, M-EA v.s. M-sEA; ns, no significant different (*P* > 0.05). Data are presented as the means ± SEM. n = 3–5 mice/group. One-way ANOVA (**B**, **C**, **E**–**I**) with Tukey’s multiple comparisons test

Therefore, we tested whether microglia are involved in this pain-anxiety comorbidity. Our analysis showed no significant difference in the number of Iba1-positive cells (a marker for microglia) or the morphology of GFAP-positive cells in the bilateral ACC between the Control and Model groups (Fig. 5A–F), indicating that astrocytes, rather than microglia, play a pivotal role in the comorbidity of pain and anxiety.

### EA inhibits the activated astrocytes in the ACC of pain-anxiety comorbidity

These data suggest that EA may alleviate pain-anxiety comorbidity and activate ACCPV by inhibiting astrocyte activation. We also discuss the effect of astrocytes on EA analgesia and anxiety relief. The area of GFAP-positive cells was significantly increased in the Model group, and EA intervention resulted in a decrease in the area of GFAP-positive cells (Fig. 5G–I). In addition, the heterogeneity of GFAP-positive cell morphology is a confounding factor that hints at astrocyte function. To address this, we conducted a Sholl analysis to measure the total lengths of branches and intersections per radius step of GFAP positive cells. In the Model group, the total length of branches, the number of branches, and the number of intersections of GFAP positive cells increased (Fig. 5J–L). However, with EA intervention, there was a decrease in the total length of branches, number of branches, and number of intersections (Fig. 5J–L). These findings suggest that EA restores the morphology and density of astrocytes in the ACC.

### Chemogenetic activating of astrocytes in the ACC reversed EA’s effect

**Fig. 6:**
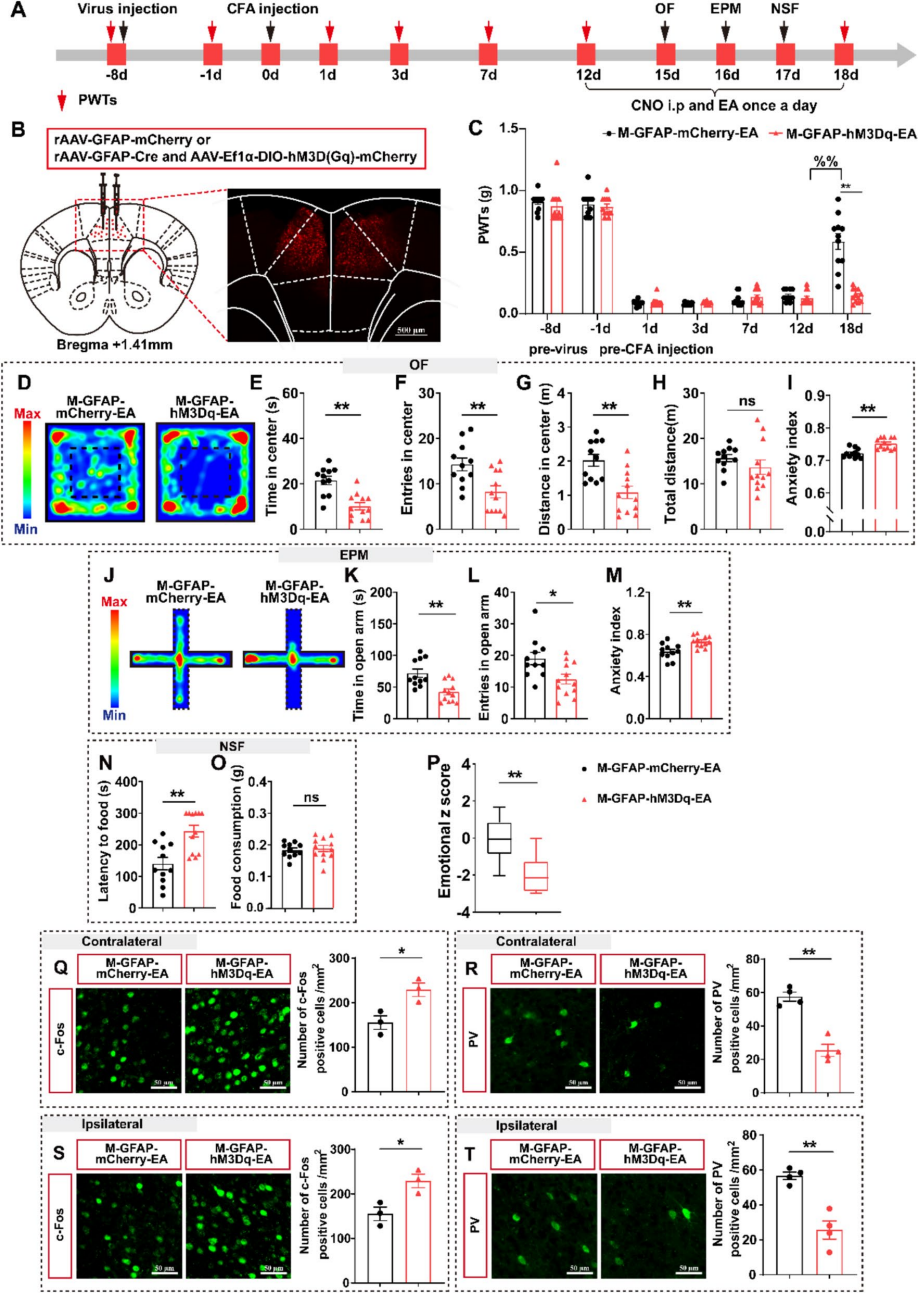
The impact of astrocytes activation on the effectiveness of EA treatment. **A** Experimental scheme of virus injection, CFA modeling, EA intervention and behavioral tests. **B** Virus injection strategy (left) and representative images show locations of virus expression (right). **C** Astrocyte activation decreased the effectiveness of EA treatment (on 18d, t = 13.28, P < 0.0001). **D**–**I** Results of OF. **D** Representative exploration traces of OF. **E** Time in center of OF (t = 4.643, P = 0.0001). **F** Entries in center of OF (t = 3.108, P = 0.0053). **G** Distance in center of OF (t = 3.687, P = 0.0014). **H** Total distance (t = 1.096, P = 0.2857). **I** Anxiety index of OF (t = 4.536, P = 0.0002). **J**–**M** Results of EPM. **J** Representative exploration traces in EPM. **K** Time in open arm of EPM (t = 3.748, P = 0.0012). **L** Entries in open arm of EPM (t = 2.672, P = 0.0143). **M** Anxiety index of EPM (t = 3.586, P = 0.0017). **N**–**O** Results of NSF. N Latency to food (t = 3.850, P = 0.0009). **M** Food consumption (t = 0.4108, P = 0.6854). **P** Emotional z score (t = 3.449, P = 0.0261). n = 11–12 mice/group. **Q** Inhibition of astrocytes activation decreased the c-Fos positive cells in contralateral ACC (t = 5.345, P < 0.0001). **R** Inhibition of astrocytes activation decreased PV positive cells in contralateral ACC (t = 7.244, P = 0.004). **S** Data representative for the c-Fos positive cells in ipsilateral ACC shown similarly to **Q** (t = 4.021, P = 0.0159). **T** Data representative for PV positive cells in ipsilateral ACC shown similarly to **R** (t = 5.523, P = 0.0015). n = 3–4 mice/group. *P < 0.05, **P < 0.01, M-GFAP-mCherry-EA v.s. M-GFAP-hM3Dq-EA; ns, no significant different (*P* > 0.05). Data are presented as the means ± SEM. Two-tailed unpaired *t*-test

To clarify the role of astrocytes in EA analgesia and emotional relief, we performed chemogenetic activation of astrocytes combined with EA and observed pain sensations and pain-related emotions (Fig. 6A, B). There were no significant differences in the PWTs of the M-GFAP-mCherry-EA and M-GFAP-hM3Dq-EA groups before CNO administration (Fig. 6C). Compared with the M-GFAP-mCherry-EA group, the PWTs of the M-GFAP-hM3Dq-EA group significantly decreased after CNO administration (Fig. 6C), suggesting that the analgesic effect of EA was reversed by astrocyte activation. Furthermore, mice in the M-GFAP-hM3Dq-EA group displayed anxiety-like behaviors in the OF, EPM, and NSF compared to the M-GFAP-mCherry-EA group, including decreased time spent, number of entries, and distance in the center area in the OF (Fig. 6D–I), decreased spent time and number of entries in the EPM (Fig. 6J–M), increased latency to food in the NSF (Fig. 6N–O), and increased anxiety index in the OF and EPM (Fig. 6I, M). Notably, there was no difference in locomotor function or food intake between the two groups (Fig. 6H and O). Moreover, the activation of astrocytic activity resulted in a decrease in the emotional z score (Fig. 6P). Additionally, we observed that the activation of astrocytic activity had an impact on both ACC neuronal activity and PV expression. Consistent with our expectations, there was an increase in c-Fos-positive cells, and a decrease in the number of PV-positive cells in the ACC of the M-GFAP-hM3Dq-EA group compared to the M-GFAP-mCherry-EA group (Fig. 6Q–T). In summary, the activation of astrocytes in the ACC reversed the effect of EA on pain-anxiety comorbidities, as well as on c-Fos and PV expression.

### Inhibition A1R mimicked the reverse effect of astrocyte activation on EA

**Fig. 7:**
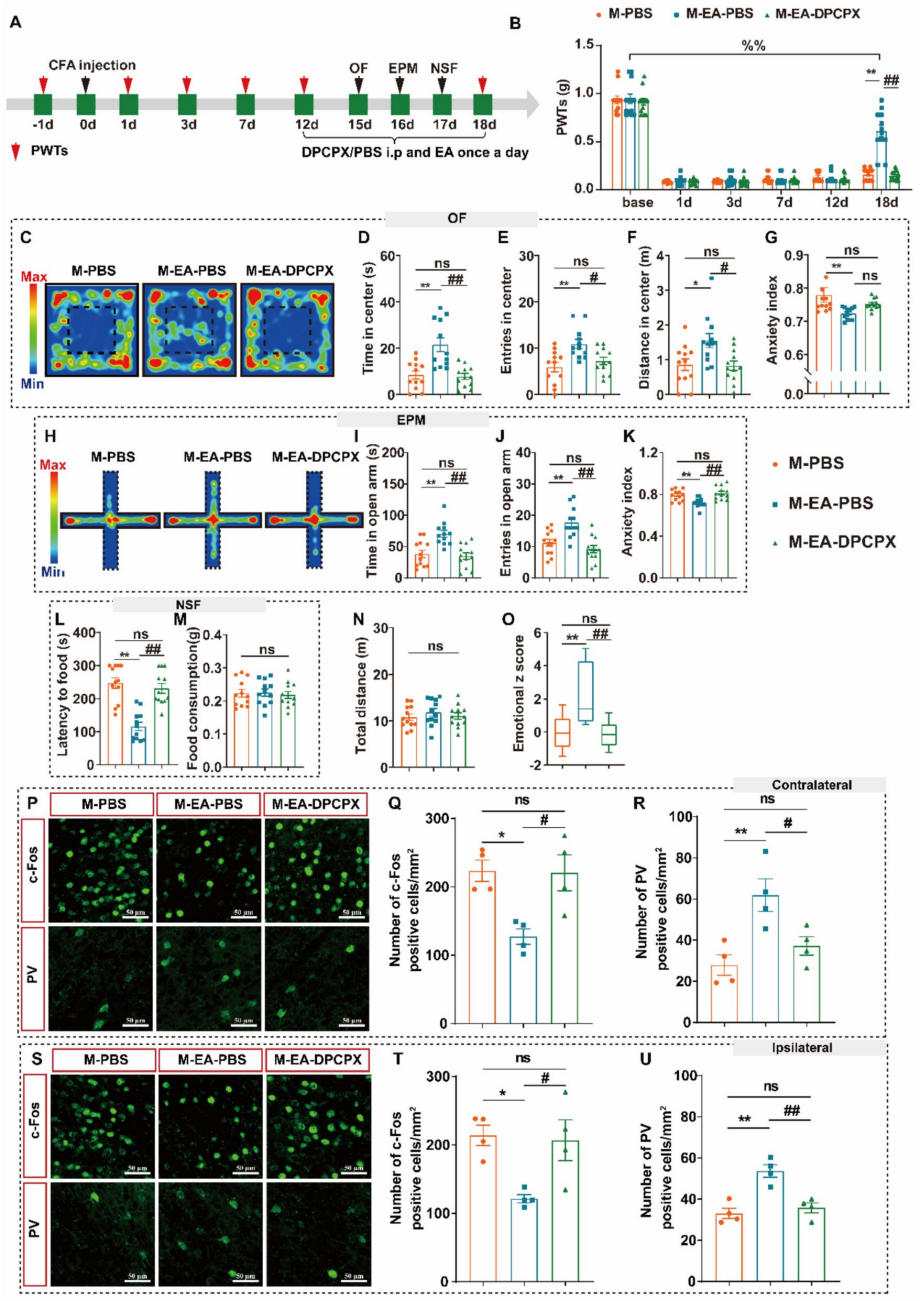
The effect of intraperitoneal injection of A1R antagonist on the therapeutic effect of EA. **A** Experimental scheme of CFA modeling, drug injection, EA intervention and behavioral tests. **B** DPCPX decreased the effect of EA on PWTs. **C**–**G** Results of OF. **C** Representative exploration traces of OF. **D** Time in center of OF (F_2,33_ = 13.33, P < 0.0001). **E** Entries in center of OF (F_2,33_ = 7.583, P = 0.0020). **F** Distance in center of OF (F_2,33_ = 5.780, P = 0.0070). **G** Anxiety index of OF (F_2,33_ = 4.242, P = 0.0199). **H**–**K** Results of EPM. **H** Representative exploration traces in EPM. **I** Time in open arm of EPM (F_2,33_ = 12.16, P = 0.0001). **J** Entries in open arm of EPM (F_2,33_ = 13.12, P < 0.0001). **K** Anxiety index of EPM (F_2,33_ = 9.437, P = 0.0006). **L**–**M** Results of NSF. L Latency to food (F_2,33_ = 224.74, P < 0.0001). **M** Food consumption (F_2,33_ = 00.1098, P = 0.88964). **N** Total distance (F_2,33_ = 0.5596, P = 0.5767). **O** Emotional z score (F_2,33_ = 13.36, P < 0.0001). **P** Representative for the c-Fos and PV positive cells in the contralateral ACC. **Q** Result of c-Fos positive cell in the contralateral ACC (F_2,9_ = 6.537, P = 0.0176). **R** Result of PV positive cell in the contralateral ACC (F_2,9_ = 8.602, P = 0.0082). **S** Representative for the c-Fos and PV positive cells in the ipsilateral ACC. **T** Date representative for c-Fos positive cells in the ipsilateral ACC shown similarly to Q (F_2,9_ = 6.912, P = 0.0152). **U** Date representative for PV positive cells in the ipsilateral ACC shown similarly to R (F_2,9_ = 17.44, P = 0.0008). *P < 0.05, **P < 0.01, M-PBS v.s. M-EA-PBS; #* P* < 0.05, ##* P* < 0.01, M-EA-PBS v.s. M-EA-DPCPX; ns, no significant different (*P* > 0.05). Data are presented as the means ± SEM. n = 11–13 mice for all mouse group. One-way ANOVA plus post hoc Tukey test

A previous study demonstrated that astrocytes participate in the regulation of contextual fear memory through adenosine A1 receptors (A1R) [53], and that local and spinal A1R are also involved in the analgesic effect of EA on pain [29, 54]. However, the role of A1R in pain comorbidities remains unclear. We observed that the protein expression level of A1R and not A2R in the ACC was decreased in model mice (Figure S7A–C), and EA intervention not only increased the A1R protein expression level (Figure S7B) but also increased the number of A1R positive cells in the ACC (Figure S7D–E). Next, we used DPCPX (i.p.), a selective A1R antagonist, combined with EA to observe PWTs and anxiety-like behaviors (Fig. 7A). Different concentrations of DPCPX reduced PWTs in control mice and injecting 0.3 mg/mL DPCPX induced the lowest PWTs. The subsequent experiments were performed at this concentration (Figure S7F). EA intervention increased the PWTs of the model mice, and DPCPX blocked the analgesic effect of EA (Fig. 7B). We also observed that DPCPX reversed the effects of EA on anxiety-like behavior in the OF, EPM, and NSF (Fig. 7C–L). DPCPX did not affect the locomotor function or feeding capacity (Fig. 7M and N). In addition, DPCPX blocked the effect of EA on the number of c-Fos and PV positive cells (Fig. 7P–U). These results suggest that the inhibition of A1R mimics the reverse effect of astrocyte activation on EA.

### A1R mediated astrocyte inhibition-induced comorbidity relief and the ACCPV enhancement

**Fig. 8:**
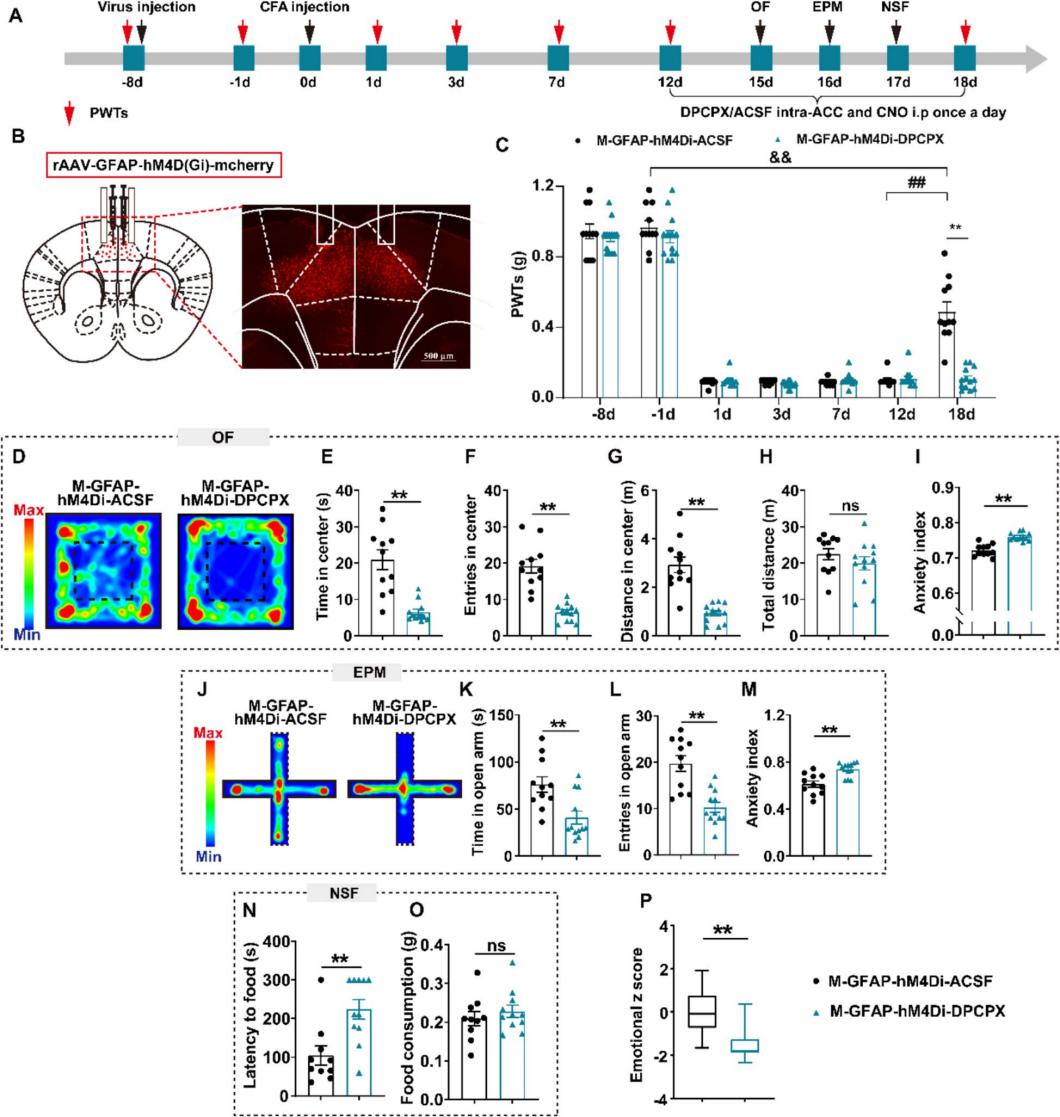
Microinjecting the A1R antagonist reversed the effect of inhibition of GFAP. **A** Experimental scheme of virus injection, CFA injection, drug administration, and behavioral tests. **B** Virus injection strategy (left) and representative image show location of virus expression (right). **C** The impact of DPCPX (i.v.) on the effect of GFAP inhibition on PWTs (on 18d, t = 12.24, P < 0.0001). **D**–**I** Results of OF. **D** Representative exploration traces of OF. **E** Time in center of OF (t = 5.223, P < 0.0001). **F** Entries in center of OF (t = 6.530, P < 0.0001). **G** Distance in center of OF (t = 6.023, P < 0.0001). **H** Total distance of OF (t = 1.044, P = 0.3083). **I** Anxiety index of OF (t = 6.508, P < 0.0001). **J**–**M** Result of EPM. **J** Representative exploration traces of EPM. **K** Time in open arm of EPM (t = 3.315, P = 0.0033). **L** Entries in open arm of EPM (t = 4.807, P < 0.0001). **M** Anxiety index of EPM (t = 4.079, *P* = 0.0005). **N**–**O** Result of NSF. **N** Latency to food (t = 3.401, *P* = 0.00330). **O** Food consumption (t = 0.77692, *P* = 0.44512). **P** Emotional z score (t = 5.902, *P* < 0.0001). ^$$^*P* < 0.01, compared with the pre-CFA injection of M-GFAP-hM4Di-ASCF; ##*P* < 0.01, compared with the 12d after CFA injection of M-GFAP-hM4Di-ASCF; **P < 0.01, M-GFAP-hM4Di-ASCF v.s. M-GFAP-hM4Di-DPCPX; ns, no significant different (*P* > 0.05). n = 10–11 mice/group. Data are presented as the means ± SEM. Two-tailed unpaired *t*-test

These results link A1R inhibition to astrocyte activation in pain-anxiety comorbidities. Therefore, we further explored whether the effect of astrocyte inhibition on relieving pain-anxiety comorbidities and increasing ACCPV required A1R (Fig. 8A–B). Before chemogenetic inhibition of astrocyte activation, DPCPX and intra-ACC decreased the PWTs (Fig. 8C–D). Unsurprisingly, DPCPX also increased anxiety-like behavior, which was decreased by inhibition of astrocyte activation (Fig. 8E–Q). In addition, intraperitoneal injection of DPCPX combined with chemogenic inhibition of astrocyte activation showed similar results (Figure S8); blocking A1R before inhibiting astrocyte activation increased the number of c-Fos-positive cells in the ACC and decreased the number of PV-positive cells in the ACC (Figure S8Q–T) and IF examination verified that A1R was co-labeled with PV (33.35%) (Figure S9). Therefore, we believe that A1R is involved in the astrocytic modulation of pain-anxiety comorbidity and PV interneuronal activity.

## Discussion

In this study, we found that EA intervention increased the PWTs, prevented anxiety-like behaviors related to inflammatory pain, enhanced PV expression, and decreased GFAP expression in the ACC. Activating ACCPV interneurons has an effect similar to that of EA on pain-anxiety comorbidity. The induction of pain- and anxiety-like behaviors was accompanied by the activation of astrocyte activity in the ACC. Inhibition of astrocyte activity prevents pain and anxiety-like behaviors and increases PV expression. Furthermore, we discovered that activation of astrocytes in the ACC reversed the effects of EA. Finally, we found that the effect of astrocytes on pain, anxiety-like behaviors, and PV interneurons was absent when A1R was inhibited. These results revealed that EA intervenes in the comorbidity of pain and anxiety by activating PV interneurons, which are modulated by the inhibition of astrocytes in the ACC, and that A1R is involved in the interaction between astrocytes and the PV interneuron.

CFA has long been used to induce local immunological responses and to cause inflammation and pain [55]. Some animal studies have focused on the acute inflammation induced by CFA intraplantar injections, typically within a week of induction. Some studies have also used the CFA model to study chronic inflammation (three or more weeks after injection) [56]. Previous studies have shown that chronic inflammation (five weeks after CFA injection) has the same effect on mechanical sensitization as acute inflammation (two days) [57]. The research also indicated that the weekly age of the mice, type of animal bedding, and concentration of CFA affect pain behavior in animal models [57, 58]. In the present study, 4 mg/mL of CFA was administered to induce prolonged inflammatory pain and anxiety-related responses. Corresponding with previous investigations, CFA administration elicited persistent inflammatory pain and anxiety-like behaviors in mice [59, 60] (Fig. 1). The effects and mechanisms of EA were investigated following the construction of a model paradigm. The analgesic efficacy of EA has been confirmed for decades [61], with a mounting body of evidence indicating EA’s regulatory impact on the comorbidity of pain and anxiety [15]. The results from these studies indicate that EA’s effect was related to parameter selection, such as acupoint combination, current density, and stimulatory frequency [62, 63]. Previous studies have demonstrated the efficacy of unilateral stimulation of the Huantiao (GB30) and Yanglingquan (GB34) acupoints in rat models of CFA administration [64]. Furthermore, EA applied to the ipsilateral GB30 and GB34 acupoints notably suppressed CFA-induced spinal microglial activation [64]. EA treatment at GB30 and GB34 exhibits a pronounced analgesic effect in rat models of chronic constriction injury (CCI) [65]. However, it remains unclear whether EA targeting GB30 and GB34 can alleviate anxiety-like behaviors associated with CFA injection. Therefore, we selected acupoint combinations based on studies conducted in our laboratory, which demonstrated that bilateral EA at ST36 effectively reduced CFA-induced inflammatory pain and alleviated the associated anxiety-like behaviors in mice and rats. Our previous research showed that 2/100 Hz EA could help manage the co-occurrence of pain and anxiety [12]. In this study, we compared the effects of EA administration at different current intensities. Previous studies have indicated that varying current intensities in EA can have distinct effects on splanchnic inflammation [66]. Surprisingly, our findings revealed that EA stimulation at 0.5 and 3 mA produced similar effects on inflammatory pain and pain-related anxiety-like behaviors (Figure S2). These results surpass our expectations and suggest that the differential mechanism by which EA mitigates visceral, and limb pain warrants further investigation in future studies. One unexpected finding was that while EA at ST36 significantly reduced the pain-anxiety comorbidity in CFA mice, it did not fully alleviate the symptoms. These outcomes suggest that EA is a beneficial, yet partial, intervention for pain-anxiety comorbidity resulting from chronic inflammation. Enhancing the efficacy of EA is the focus of ongoing research. In addition, many researchers are interested in whether restraint affects mouse behavior, as previous studies have shown that prolonged immobilization can induce stress in mice [67]. In this study, all mice were lightly restrained, but not tightly immobilized. Mice in the control group did not exhibit any signs of anxiety-like behavior. These findings suggested that light restraint does not induce stress in mice. Prior research involving loosely restrained rats during EA application also demonstrated that this method did not alter the effectiveness of EA [12]. Collectively, these findings indicated that the efficacy of EA was not influenced by light restraint.

It has been reported that ACC plays an important role in EA modulation of pain and pain-related emotion [14, 68]. We have shown that the GABAergic system reduces inhibitory presynaptic transmission in the ACC, enhancing pyramidal neuron excitability and contributing to chronic inflammatory pain-related anxiety [69]. Furthermore, we demonstrated that EA alleviates chronic inflammatory pain and pain-related anxiety by selectively upregulating PV-but not SOM-expressing interneurons in the ACC. Chemogenetic inhibition of PV interneurons abolishing EA’s beneficial effects on pain and emotion regulation [12]. PV interneurons are the most abundant GABAergic interneurons, accounting for approximately 40% of GABAergic interneurons [70]. While previous studies have reported that PV interneurons are important modulators in the pathophysiology of anxiety disorders and chronic pain [17, 71], the present results suggest that the activation of ACCPV has a similar effect to that of EA on pain and pain-anxiety behavior. However, the underlying mechanism by which EA upregulates the expression of ACCPV to alleviate pain-anxiety comorbidity remains unclear.

Nonneuronal cells, particularly glial cells, play an important role in chronic pain [72]. More than half of the cells in the central nervous system are glia [73]. Astrocytes and microglia are the two types of glial cells. Their activation plays an important role in chronic pain, although they play different roles in the induction and persistence of chronic pain [50]. Although spinal astrocytes contribute to almost all persistent pain conditions, spinal microglia are involved only in regulating specific types of pain [74]. For instance, nerve injury leads to the activation of both microglia and astrocytes, whereas chemotherapy-induced peripheral neuropathy (CIPN) in rats is associated with significant astrogliosis but minimal microgliosis [75]. At the supraspinal level, we also observed that astrocytes, but not microglia, in the ACC with chronic inflammatory pain-anxiety comorbidity were overactivated and exhibited morphological changes such as hypertrophy and proliferation, indicating that astrocytes in the ACC are more correlated with pain-anxiety comorbidity than microglia in this state, which is consistent with previous findings that astrocytes in the ACC play a role in the affective component of pain, which encompasses unpleasantness or aversion [22]. In a neuropathic pain model, EA markedly decreased GFAP expression in the amygdala, relieved neuropathic pain, and reduced comorbid negative behavior [76]. In our study, we also found that EA decreased GFAP expression in the ACC and changed the heterogeneity of its morphology, including decreasing the total length of branches, number of branches, and number of intersections. Activation of astrocytes in the ACC reserved the effect of EA on pain-anxiety comorbidities and PV-positive cells. Therefore, we believe that EA intervenes in the comorbidity of pain and anxiety by inhibiting astrocytes in the ACC. Furthermore, by modulating astrocyte activation in the ACC, we not only reserved (Fig. 3) or induced (Fig. 4) pain-anxiety comorbidity behaviors in mice and altered the expression levels of c-Fos and PV in the ACC but also impaired the efficacy of EA on mouse behaviors and c-Fos and PV expression. These findings establish a connection between the impact of EA on PV, pain-anxiety comorbidity, and astrocyte activation. Our findings suggest that EA enhances PV function in the ACC, thereby alleviating pain and anxiety-like behaviors by inhibiting astrocyte activation.

In this study, we investigated the substance that links astrocytes and PV interneurons, which may be involved in EA’s effect. Many studies have suggested that astrocytes participate in complex operational models of cortical networks, modulate different brain rhythms, and import synaptic transmission and plasticity from pyramidal cells and GABAergic interneurons [31]. In particular, astrocytes regulated the activity of PV interneurons [77, 78]. These actions can be achieved by the release of gliotransmitters (such as ATP and adenosine), which activate pre-and/or postsynaptic receptors (adenosine 1 receptor (A1R) or adenosine 2 receptor (A2R)) in neurons [79, 80]. Local A1R is involved in the analgesic effect of acupuncture, and direct acupoint ST36 injection of an A1R agonist replicates the analgesic effect of acupuncture/EA on inflammatory pain [29]. Direct injection of A1R antagonists into the local acupoint ST36 significantly impaired EA-induced analgesia in CFA rats [81]. Another study reported that A1R is involved in the action of EA during neuropathic pain by suppressing spinal astrocyte activation [82]. In this study, we expanded our findings by demonstrating that the effect of EA on PV interneurons can be blocked by the A1R antagonist DPCPX, whether administered intraperitoneally or via intraventricular microinjection (Fig. 7). Additionally, we showed that blocking the A1R reversed the influence of astrocyte deactivation, a crucial element of EA in the ACC, on PV interneurons and mouse behavior. Hence, we propose that A1R plays a role in the regulatory effects of EA on PV interneurons in the context of pain-anxiety comorbidity. Notably, the involvement of A1R in EA treatment exceeded our initial expectations. We initially hypothesized that EA would activate PV interneurons by reducing adenosine release or deactivating A1R, because astrocyte activation leads to increased adenosine release and EA deactivates astrocytes in the ACC. However, our results indicate that PV interneurons may be activated by A1R stimulation. Previous studies demonstrated that ATP and adenosine derived from astrocytes have different effects on different neurons and neural circuits [80, 83]. For example, hippocampal astrocyte derived ATP and adenosine downregulate excitatory synaptic transmission by activating the presynaptic A1R and enhance inhibitory synaptic transmission by activating the postsynaptic P2Y1 receptor, thus effectively downregulating the excitability of the entire hippocampal neural circuit [83]. Downregulation of excitatory synaptic transmission by different subtypes of excitatory interneurons contributes to heterosynaptic inhibitory effects in the hippocampus. Adenosine derived from the central amygdala (CeA) astrocytes inhibits excitatory synaptic transmission by activating presynaptic adenosine A1 receptors while enhancing inhibitory synaptic transmission by activating adenosine A2A receptors, thereby reducing the excitability of CeA neurons [80]. ChR2 activates astrocytes in the visual cortex, boosts the firing rate of PV interneurons, and affects visual response properties [34]. Research has also reported that GABA interneurons express A1R receptors and that their excitability decreases after ATP or adenosine stimulation [38, 84]. Furthermore, our results revealed that only 33.5% of A1R were co-labeled with PV interneurons. This suggests the existence of a microcircuit that regulates the function of PV interneurons in the ACC, including adenosine and A1R. Further investigation is required to elucidate the specificity of this interaction.

This study has some limitations. First, only male mice were used to investigate the underlying mechanisms of EA to address the comorbidity of pain and anxiety. Previous studies have reported conflicting results regarding sex differences in pain and analgesia in mouse models, underscoring the importance of considering sex as a variable [85–88]. Moreover, sex disparities in the analgesic effects of EA remain unclear. Smeester et al. [89] focused on sex-specific responses to EA treatment in rodent models of chronic pain. They administered EA at the ST 36 acupoint biweekly to alleviate hyperalgesia and found that males experienced earlier onset of analgesia, while females exhibited prolonged relief. Our current investigation aimed to evaluate the impact of EA on comorbid pain and anxiety behaviors in male mice. Second, we only examined microglial quantity without investigating their functional state or specific mechanisms linking microglia to the comorbidity of pain and anxiety. Further work should employ more refined techniques to dissect microglial activation patterns and their functional contributions to this comorbidity. Third, it should be clarified whether the gliotransmitters released by astrocytes in the ACC increase with the comorbidity of pain, anxiety, and EA regulation effects. Fourth, further studies are needed to elucidate the contribution of A1R in the ACC to the effects of EA in A1R knockout animals. Finally, the role of A1R in the EA regulation of astrocytes and PV interneurons in the ACC has not been clarified.

## Conclusions

In conclusion, the present results provide direct evidence that activated astrocytes inhibit the function of PV interneurons, resulting in the development of the comorbidities of pain and anxiety, which depend on the involvement of A1R. EA alleviates pain and anxiety-like behavior induced by CFA by activating PV interneurons, modulated by the inhibition of astrocytes in the ACC.

## Supplementary Information

Additional file 1.

## Data Availability

The key data are contained in the figures, tables, and additional files. The data sets used and/or analyzed during this study can be further obtained from the corresponding author on reasonable request.

## Abbreviations

A1R: Adenosine 1 receptor
A2R: Adenosine 2 receptor
ACC: Anterior cingulate cortex
CeA: Central amygdala
CFA: Complete Freund’s adjuvant
CIPN: Chemotherapy induced peripheral neuropathy
CNS: Central nervous system
EA: Electroacupuncture
EPM: Elevated plus maze
GABA: γ-Aminobutyric acid
NSF: Novelty-suppressed feeding
OF: Open field
PBS: Phosphate buffered saline
PFA: Paraformaldehyde
PV: Parvalbumin
PWTs: Paw withdrawal thresholds

## References

[1] Cohen SP, Vase L, Hooten WM. Chronic pain: an update on burden, best practices, and new advances. Lancet. 2021;397:2082–97. 34062143 10.1016/S0140-6736(21)00393-7

[2] Velly AM, Mohit S. Epidemiology of pain and relation to psychiatric disorders. Prog Neuropsychopharmacol Biol Psychiatry. 2018;87:159–67. 28522289 10.1016/j.pnpbp.2017.05.012

[3] Choi KW, Kim YK, Jeon HJ. Comorbid anxiety and depression: clinical and conceptual consideration and transdiagnostic treatment. Adv Exp Med Biol. 2020;1191:219–35. 32002932 10.1007/978-981-32-9705-0_14

[4] Chen T, Wang J, Wang YQ, Chu YX. Current understanding of the neural circuitry in the comorbidity of chronic pain and anxiety. Neural Plast. 2022;2022:4217593. 35211169 10.1155/2022/4217593 PMC8863453

[5] Zhuo M. Neural mechanisms underlying anxiety-chronic pain interactions. Trends Neurosci. 2016;39:136–45. 26878750 10.1016/j.tins.2016.01.006

[6] Hartz SM, Culverhouse RC, Mintz CM, Ellis MS, Kasper ZA, Cavazos-Rehg P, et al. Association between recent overdose and chronic pain among individuals in treatment for opioid use disorder. PLoS ONE. 2022;17:e0271379. 36441691 10.1371/journal.pone.0271379 PMC9704550

[7] Hobelmann JG, Huhn AS. Comprehensive pain management as a frontline treatment to address the opioid crisis. Brain Behav. 2021;11:e2369. 34555260 10.1002/brb3.2369 PMC8613403

[8] Vickers AJ, Vertosick EA, Lewith G, MacPherson H, Foster NE, Sherman KJ, et al. Acupuncture for chronic pain: update of an individual patient data meta-analysis. J Pain. 2018;19:455–74. 29198932 10.1016/j.jpain.2017.11.005 PMC5927830

[9] You J, Li H, Xie D, Chen R, Chen M. Acupuncture for chronic pain-related depression: a systematic review and meta-analysis. Pain Res Manag. 2021;2021:6617075. 33680223 10.1155/2021/6617075 PMC7925064

[10] Chen T, Zhang WW, Chu YX, Wang YQ. Acupuncture for pain management: molecular mechanisms of action. Am J Chin Med. 2020;48:793–811. 32420752 10.1142/S0192415X20500408

[11] Chen Y, Tong S, Xu Y, Xu Y, Wu Z, Zhu X, et al. Involvement of basolateral amygdala-rostral anterior cingulate cortex in mechanical allodynia and anxiety-like behaviors and potential mechanisms of electroacupuncture. CNS Neurosci Ther. 2024;30(9):e70035. 39279046 10.1111/cns.70035 PMC11402788

[12] Shao F, Fang J, Qiu M, Wang S, Xi D, Shao X, et al. Electroacupuncture ameliorates chronic inflammatory pain-related anxiety by activating PV interneurons in the anterior cingulate cortex. Front Neurosci. 2021;15:691931. 34290586 10.3389/fnins.2021.691931 PMC8287862

[13] Du J, Fang J, Xu Z, Xiang X, Wang S, Sun H, et al. Electroacupuncture suppresses the pain and pain-related anxiety of chronic inflammation in rats by increasing the expression of the NPS/NPSR system in the ACC. Brain Res. 2020;1733:146719. 32044336 10.1016/j.brainres.2020.146719

[14] Xu Z, Fang J, Xiang X, Sun H, Wang S, Fang J, et al. Electroacupuncture alleviates pain-related emotion by upregulating the expression of NPS and Its receptor NPSR in the anterior cingulate cortex and hypothalamus. Evid Based Complement Alternat Med. 2020;2020:8630368. 32104195 10.1155/2020/8630368 PMC7035524

[15] Wu Z, Shen Z, Xu Y, Chen S, Xiao S, Ye J, et al. A neural circuit associated with anxiety-like behaviors induced by chronic inflammatory pain and the anxiolytic effects of electroacupuncture. CNS Neurosci Ther. 2024;30:e14520. 38018559 10.1111/cns.14520 PMC11017463

[16] Li QY, Duan YW, Zhou YH, Chen SX, Li YY, Zang Y. NLRP3-Mediated Piezo1 upregulation in ACC inhibitory parvalbumin-expressing interneurons is involved in pain processing after peripheral nerve injury. Int J Mol Sci. 2022;23:13035. 36361825 10.3390/ijms232113035 PMC9655876

[17] Kang SJ, Kwak C, Lee J, Sim SE, Shim J, Choi T, et al. Bidirectional modulation of hyperalgesia via the specific control of excitatory and inhibitory neuronal activity in the ACC. Mol Brain. 2015;8(1):81. 26631249 10.1186/s13041-015-0170-6 PMC4668615

[18] Jiang J, Tan S, Feng X, Peng Y, Long C, Yang L. Distinct ACC neural mechanisms underlie authentic and transmitted anxiety induced by maternal separation in mice. J Neurosci. 2023;43:8201–18. 37845036 10.1523/JNEUROSCI.0558-23.2023 PMC10697407

[19] Losi G, Mariotti L, Carmignoto G. Gabaergic interneuron to astrocyte signalling: a neglected form of cell communication in the brain. Philos Trans R Soc Lond B Biol Sci. 2014;369:20130609. 25225102 10.1098/rstb.2013.0609 PMC4173294

[20] Masocha W. Astrocyte activation in the anterior cingulate cortex and altered glutamatergic gene expression during paclitaxel-induced neuropathic pain in mice. PeerJ. 2015;3:e1350. 26528412 10.7717/peerj.1350 PMC4627912

[21] Wang J, Tu J, Cao B, Mu L, Yang X, Cong M, et al. Astrocytic l-lactate signaling facilitates amygdala-anterior cingulate cortex synchrony and decision making in rats. Cell Rep. 2017;21:2407–18. 29186680 10.1016/j.celrep.2017.11.012

[22] Chen FL, Dong YL, Zhang ZJ, Cao DL, Xu J, Hui J, et al. Activation of astrocytes in the anterior cingulate cortex contributes to the affective component of pain in an inflammatory pain model. Brain Res Bull. 2012;87:60–6. 22004615 10.1016/j.brainresbull.2011.09.022

[23] Takeda I, Yoshihara K, Cheung DL, Kobayashi T, Agetsuma M, Tsuda M, et al. Controlled activation of cortical astrocytes modulates neuropathic pain-like behaviour. Nat Commun. 2022;13:4100. 35835747 10.1038/s41467-022-31773-8 PMC9283422

[24] Blaszczyk L, Maître M, Lesté-Lasserre T, Clark S, Cota D, Oliet SHR, et al. Sequential alteration of microglia and astrocytes in the rat thalamus following spinal nerve ligation. J Neuroinflammation. 2018;15:349. 30572902 10.1186/s12974-018-1378-z PMC6302506

[25] Burke NN, Geoghegan E, Kerr DM, Moriarty O, Finn DP, Roche M. Altered neuropathic pain behaviour in a rat model of depression is associated with changes in inflammatory gene expression in the amygdala. Genes Brain Behav. 2013;12:705–13. 23957449 10.1111/gbb.12080

[26] Araque A, Carmignoto G, Haydon PG, Oliet SH, Robitaille R, Volterra A. Gliotransmitters travel in time and space. Neuron. 2014;81:728–39. 24559669 10.1016/j.neuron.2014.02.007 PMC4107238

[27] Stogsdill JA, Eroglu C. The interplay between neurons and glia in synapse development and plasticity. Curr Opin Neurobiol. 2017;42:1–8. 27788368 10.1016/j.conb.2016.09.016 PMC5316301

[28] Kim NS, Chung WS. Astrocytes regulate neuronal network activity by mediating synapse remodeling. Neurosci Res. 2023;187:3–13. 36170922 10.1016/j.neures.2022.09.007

[29] Goldman N, Chen M, Fujita T, Xu Q, Peng W, Liu W, et al. Adenosine A1 receptors mediate local anti-nociceptive effects of acupuncture. Nat Neurosci. 2010;13:883–8. 20512135 10.1038/nn.2562 PMC3467968

[30] Yin HY, Fan YP, Liu J, Li DT, Guo J, Yu SG. Purinergic ATP triggers moxibustion-induced local anti-nociceptive effect on inflammatory pain model. Purinergic Signal. 2023;19:5–12. 34378078 10.1007/s11302-021-09815-5 PMC9984580

[31] Mederos S, Perea G. GABAergic-astrocyte signaling: a refinement of inhibitory brain networks. Glia. 2019;67:1842–51. 31145508 10.1002/glia.23644 PMC6772151

[32] Mederos S, Sánchez-Puelles C, Esparza J, Valero M, Ponomarenko A, Perea G. Gabaergic signaling to astrocytes in the prefrontal cortex sustains goal-directed behaviors. Nat Neurosci. 2021;24:82–92. 33288910 10.1038/s41593-020-00752-x

[33] Wahis J, Hennes M, Arckens L, Holt MG. Star power: the emerging role of astrocytes as neuronal partners during cortical plasticity. Curr Opin Neurobiol. 2021;67:174–82. 33360483 10.1016/j.conb.2020.12.001 PMC8202513

[34] Perea G, Yang A, Boyden ES, Sur M. Optogenetic astrocyte activation modulates response selectivity of visual cortex neurons in vivo. Nat Commun. 2014;5:3262. 24500276 10.1038/ncomms4262 PMC4075037

[35] Chen YH, Xie SY, Chen CW, Lu DY. Electroacupuncture improves repeated social defeat stress-elicited social avoidance and anxiety-like behaviors by reducing Lipocalin-2 in the hippocampus. Mol Brain. 2021;14:150. 34565419 10.1186/s13041-021-00860-0 PMC8474847

[36] Xie L, Liu Y, Zhang N, Li C, Sandhu AF, Williams G3rd, et al. Electroacupuncture improves M2 microglia polarization and glia anti-inflammation of hippocampus in Alzheimer’s disease. Front Neurosci. 2021;15:689629. 34646113 10.3389/fnins.2021.689629 PMC8502881

[37] Ochiishi T, Chen L, Yukawa A, Saitoh Y, Sekino Y, Arai T, et al. Cellular localization of adenosine A1 receptors in rat forebrain: immunohistochemical analysis using adenosine A1 receptor-specific monoclonal antibody. J Comp Neurol. 1999;411:301–16. 10404255

[38] Yang C, Franciosi S, Brown RE. Adenosine inhibits the excitatory synaptic inputs to Basal forebrain cholinergic, GABAergic, and parvalbumin neurons in mice. Front Neurol. 2013;4:77. 23801984 10.3389/fneur.2013.00077 PMC3687201

[39] Liao HY, Hsieh CL, Huang CP, Lin YW. Electroacupuncture attenuates CFA-induced inflammatory pain by suppressing Nav1.8 through S100B, TRPV1, opioid, and adenosine pathways in mice. Sci Rep. 2017;7:42531. 28211895 10.1038/srep42531 PMC5304170

[40] Wu M, Chen Y, Shen Z, Zhu Y, Xiao S, Zhu X, et al. Electroacupuncture alleviates anxiety-like behaviors induced by chronic neuropathic pain via regulating different dopamine receptors of the basolateral amygdala. Mol Neurobiol. 2022;59:5299–311. 35696012 10.1007/s12035-022-02911-6 PMC9395447

[41] Chaplan SR, Bach FW, Pogrel JW, Chung JM, Yaksh TL. Quantitative assessment of tactile allodynia in the rat paw. J Neurosci Methods. 1994;53:55–63. 7990513 10.1016/0165-0270(94)90144-9

[42] Fotio Y, Jung KM, Palese F, Obenaus A, Tagne AM, Lin L, et al. NAAA-regulated lipid signaling governs the transition from acute to chronic pain. Sci Adv. 2021;7:eabi8834. 34678057 10.1126/sciadv.abi8834 PMC8535814

[43] Yamauchi N, Sato K, Sato K, Murakawa S, Hamasaki Y, Nomura H, et al. Chronic pain-induced neuronal plasticity in the bed nucleus of the stria terminalis causes maladaptive anxiety. Sci Adv. 2022;8:eabj5586. 35476439 10.1126/sciadv.abj5586 PMC9045713

[44] Codeluppi SA, Xu M, Bansal Y, Lepack AE, Duric V, Chow M, et al. Prefrontal cortex astroglia modulate anhedonia-like behavior. Mol Psychiatry. 2023;28:4632–41. 37696873 10.1038/s41380-023-02246-1 PMC10914619

[45] Sherathiya VN, Schaid MD, Seiler JL, Lopez GC, Lerner TN. GuPPy, a Python toolbox for the analysis of fiber photometry data. Sci Rep. 2021;11:24212. 34930955 10.1038/s41598-021-03626-9 PMC8688475

[46] Schindelin J, Arganda-Carreras I, Frise E, Kaynig V, Longair M, Pietzsch T, et al. Fiji: an open-source platform for biological-image analysis. Nat Methods. 2012;9(7):676–82. 22743772 10.1038/nmeth.2019 PMC3855844

[47] Yang L, Lu J, Guo J, Chen J, Xiong F, Wang X, et al. Ventrolateral periaqueductal gray astrocytes regulate nociceptive sensation and emotional motivation in diabetic neuropathic pain. J Neurosci. 2022;42(43):8184–99. 36109166 10.1523/JNEUROSCI.0920-22.2022 PMC9636999

[48] Ferreira TA, Blackman AV, Oyrer J, Jayabal S, Chung AJ, Watt AJ, et al. Neuronal morphometry directly from bitmap images. Nat Methods. 2014;11:982–4. 25264773 10.1038/nmeth.3125 PMC5271921

[49] Yang L, Qi Y, Yang Y. Astrocytes control food intake by inhibiting AGRP neuron activity via adenosine A1 receptors. Cell Rep. 2015;11:798–807. 25921535 10.1016/j.celrep.2015.04.002

[50] Ji RR, Donnelly CR, Nedergaard M. Astrocytes in chronic pain and itch. Nat Rev Neurosci. 2019;20(11):667–85. 31537912 10.1038/s41583-019-0218-1 PMC6874831

[51] Zhang Y, Guo Z, Yang L, Cheng C, Gai C, Gao Y, et al. Possible involvement of perineuronal nets in anti-depressant effects of electroacupuncture in chronic-stress-induced depression in rats. Neurochem Res. 2023;48(10):3146–59. 37347359 10.1007/s11064-023-03970-4

[52] Yuan S, Qiu B, Liang Y, Deng B, Xu J, Tang X, et al. Role of TRPV1 in electroacupuncture-mediated signal to the primary sensory cortex during regulation of the swallowing function. CNS Neurosci Ther. 2024;30:e14457. 37718934 10.1111/cns.14457 PMC10916430

[53] Li Y, Li L, Wu J, Zhu Z, Feng X, Qin L, et al. Activation of astrocytes in hippocampus decreases fear memory through adenosine A1 receptors. Elife. 2020;9:e57155. 32869747 10.7554/eLife.57155 PMC7505657

[54] Dai QX, Huang LP, Mo YC, Yu LN, Du WW, Zhang AQ, et al. Role of spinal adenosine A1 receptors in the analgesic effect of electroacupuncture in a rat model of neuropathic pain. J Int Med Res. 2020;48:300060519883748. 31868057 10.1177/0300060519883748 PMC7783270

[55] Banik RK, Sia T, Ibrahim MM, Sivanesan E, Uhelski M, Pena A, et al. Increases in local skin temperature correlate with spontaneous foot lifting and heat hyperalgesia in both incisional inflammatory models of pain. Pain Rep. 2023;8:e1097. 37711430 10.1097/PR9.0000000000001097 PMC10499105

[56] Whitehouse MW. Adjuvant arthritis 50 years on: The impact of the 1956 article by C. M. Pearson, “Development of arthritis, periarthritis and periostitis in rats given adjuvants.” Inflamm Res. 2007;56:133–8. 17522809 10.1007/s00011-006-6117-8

[57] Moehring F, O’Hara CL, Stucky CL. Bedding material affects mechanical thresholds, heat thresholds, and texture preference. J Pain. 2016;17:50–64. 26456764 10.1016/j.jpain.2015.08.014 PMC4698037

[58] Garrison SR, Stucky CL. Contribution of transient receptor potential ankyrin 1 to chronic pain in aged mice with complete freund’s adjuvant-induced arthritis. Arthritis Rheumatol. 2014;66:2380–90. 24891324 10.1002/art.38724 PMC4149259

[59] Hong J, Li JN, Wu FL, Bao SY, Sun HX, Zhu KH, et al. Projections from anteromedial thalamus nucleus to the midcingulate cortex mediate pain and anxiety-like behaviors in mice. Neurochem Int. 2023;171:105640. 37951541 10.1016/j.neuint.2023.105640

[60] Li YJ, Du WJ, Liu R, Zan GY, Ye BL, Li Q, et al. Paraventricular nucleus-central amygdala oxytocinergic projection modulates pain-related anxiety-like behaviors in mice. CNS Neurosci Ther. 2023;29:3493–506. 37248645 10.1111/cns.14282 PMC10580334

[61] Guo Y, Li Y, Xu T, Zhu M, Xu Z, Dou B, et al. An inspiration to the studies on mechanisms of acupuncture and moxibustion action derived from 2021 Nobel Prize in physiology or medicine. Acupunct Herbal Med. 2022;2:1–8.

[62] Li B, Zhang M, Ngaenklangdon S, Jiang H, Zhu W, Zhuo B, et al. How to conduct an acupuncture dose–effect relationship study? A discussion based on study methodology. Acupunct Herbal Med. 2022;2:221–8.

[63] Guo Z, Wei N, Ye R, Sun T, Qiu S, et al. Map activation of various brain regions using different frequencies of electroacupuncture ST36, utilizing the FosCreER strategy. Acupunct Herb Med. 2024;4:386–98.

[64] Shan S, Qi-Liang MY, Hong C, Tingting L, Mei H, Haili P, et al. Is functional state of spinal microglia involved in the anti-allodynic and anti-hyperalgesic effects of electroacupuncture in rat model of monoarthritis? Neurobiol Dis. 2007;26:558–68. 17442579 10.1016/j.nbd.2007.02.007 PMC2681292

[65] Jiang M, Chen X, Zhang L, Liu W, Yu X, Wang Z, et al. Electroacupuncture suppresses glucose metabolism and GLUT-3 expression in medial prefrontal cortical in rats with neuropathic pain. Biol Res. 2021;54:24. 34362470 10.1186/s40659-021-00348-0 PMC8344173

[66] Liu S, Wang ZF, Su YS, Ray RS, Jing XH, Wang YQ, et al. Somatotopic organization and intensity dependence in driving distinct NPY-expressing sympathetic pathways by electroacupuncture. Neuron. 2020;108:436-450.e7. 32791039 10.1016/j.neuron.2020.07.015 PMC7666081

[67] Kim KS, Han PL. Optimization of chronic stress paradigms using anxiety- and depression-like behavioral parameters. J Neurosci Res. 2006;83:497–507. 16416425 10.1002/jnr.20754

[68] Du J, Fang J, Wen C, Shao X, Liang Y, Fang J. The effect of electroacupuncture on PKMzeta in the ACC in regulating anxiety-like behaviors in rats experiencing chronic inflammatory pain. Neural Plast. 2017;2017:3728752. 29075535 10.1155/2017/3728752 PMC5624165

[69] Shao FB, Fang JF, Wang SS, Qiu MT, Xi DN, Jin XM, et al. Anxiolytic effect of GABAergic neurons in the anterior cingulate cortex in a rat model of chronic inflammatory pain. Mol Brain. 2021;10(14):139. 10.1186/s13041-021-00849-9 PMC8431944 34507588

[70] DeFelipe J, López-Cruz PL, Benavides-Piccione R, Bielza C, Larrañaga P, Anderson S, et al. New insights into the classification and nomenclature of cortical GABAergic interneurons. Nat Rev Neurosci. 2013;14:202–16. 23385869 10.1038/nrn3444 PMC3619199

[71] Luo ZY, Huang L, Lin S, Yin YN, Jie W, Hu NY, et al. Erbin in amygdala parvalbumin-positive neurons modulates anxiety-like behaviors. Biol Psychiatry. 2020;87:926–36. 31889536 10.1016/j.biopsych.2019.10.021

[72] Ji RR, Chamessian A, Zhang YQ. Pain regulation by non-neuronal cells and inflammation. Science. 2016;354:572–7. 27811267 10.1126/science.aaf8924 PMC5488328

[73] Donnelly CR, Andriessen AS, Chen G, Wang K, Jiang C, Maixner W, et al. Central nervous system targets: glial cell mechanisms in chronic pain. Neurotherapeutics. 2020;17:846–60. 32820378 10.1007/s13311-020-00905-7 PMC7609632

[74] Ji RR, Berta T, Nedergaard M. Glia and pain: is chronic pain a gliopathy? Pain. 2013;154(Suppl 1):S10–28. 23792284 10.1016/j.pain.2013.06.022 PMC3858488

[75] Robinson CR, Zhang H, Dougherty PM. Astrocytes, but not microglia, are activated in oxaliplatin and bortezomib-induced peripheral neuropathy in the rat. Neuroscience. 2014;274:308–17. 24905437 10.1016/j.neuroscience.2014.05.051 PMC4099296

[76] Zhang XH, Feng CC, Pei LJ, Zhang YN, Chen L, Wei XQ, et al. Electroacupuncture attenuates neuropathic pain and comorbid negative behavior: the involvement of the dopamine system in the amygdala. Front Neurosci. 2021;15:657507. 34025342 10.3389/fnins.2021.657507 PMC8137986

[77] Losi G, Lia AM, Gomez-Gonzalo M, Zonta M, Carmignoto G. Optogenetic interneuron stimulation and calcium imaging in astrocytes. Methods Mol Biol. 2019;1925:173–82. 30674027 10.1007/978-1-4939-9018-4_16

[78] Mariotti L, Losi G, Lia A, Melone M, Chiavegato A, Gómez-Gonzalo M, et al. Interneuron-specific signaling evokes distinctive somatostatin-mediated responses in adult cortical astrocytes. Nat Commun. 2018;9:82. 29311610 10.1038/s41467-017-02642-6 PMC5758790

[79] Panatier A, Vallée J, Haber M, Murai KK, Lacaille JC, Robitaille R. Astrocytes are endogenous regulators of basal transmission at central synapses. Cell. 2011;146:785–98. 21855979 10.1016/j.cell.2011.07.022

[80] Martin-Fernandez M, Jamison S, Robin LM, Zhao Z, Martin ED, Aguilar J, et al. Synapse-specific astrocyte gating of amygdala-related behavior. Nat Neurosci. 2017;20:1540–8. 28945222 10.1038/nn.4649 PMC5903286

[81] Zhang RY, Zhu BF, Wang LK, Song Y, Zhao JG, Guo Y, et al. Electroacupuncture alleviates inflammatory pain via adenosine suppression and its mediated substance P expression. Arq Neuropsiquiatr. 2020;78:617–23. 33146290 10.1590/0004-282X20200078

[82] Zhang M, Dai Q, Liang D, Li D, Chen S, Chen S, et al. Involvement of adenosine A1 receptor in electroacupuncture-mediated inhibition of astrocyte activation during neuropathic pain. Arq Neuropsiquiatr. 2018;76:736–42. 30570016 10.1590/0004-282X20180128

[83] Tonazzini I, Trincavelli ML, Storm-Mathisen J, Martini C, Bergersen LH. Co-localization and functional cross-talk between A1 and P2Y1 purine receptors in rat hippocampus. Eur J Neurosci. 2007;26:890–902. 17672857 10.1111/j.1460-9568.2007.05697.x PMC2121138

[84] Matos M, Bosson A, Riebe I, Reynell C, Vallée J, Laplante I, et al. Astrocytes detect and upregulate transmission at inhibitory synapses of somatostatin interneurons onto pyramidal cells. Nat Commun. 2018;9:4254. 30315174 10.1038/s41467-018-06731-y PMC6185912

[85] Fabris D, Carvalho MC, Brandão ML, Prado WA, Zuardi AW, Crippa JA, et al. Sex-dependent differences in the anxiolytic-like effect of cannabidiol in the elevated plus-maze. J Psychopharmacol. 2022;36:1371–83. 36239039 10.1177/02698811221125440 PMC9716492

[86] Bryant CD, Eitan S, Sinchak K, Fanselow MS, Evans CJ. NMDA receptor antagonism disrupts the development of morphine analgesic tolerance in male, but not female C57BL/6J mice. Am J Physiol Regul Integr Comp Physiol. 2006;291:R315–26. 16601258 10.1152/ajpregu.00831.2005

[87] Juni A, Klein G, Kowalczyk B, Ragnauth A, Kest B. Sex differences in hyperalgesia during morphine infusion: effect of gonadectomy and estrogen treatment. Neuropharmacology. 2008;54:1264–70. 18457849 10.1016/j.neuropharm.2008.04.004

[88] Rasakham K, Liu-Chen LY. Sex differences in kappa opioid pharmacology. Life Sci. 2011;88:2–16. 20951148 10.1016/j.lfs.2010.10.007 PMC3870184

[89] Smeester BA, Al-Gizawiy M, Beitz AJ. Effects of different electroacupuncture scheduling regimens on murine bone tumor-induced hyperalgesia: sex differences and role of inflammation. Evid Based Complement Alternat Med. 2012;2012:671386. 23320035 10.1155/2012/671386 PMC3541553

