# Supplementary material for: Astrocyte inhibition and PV interneuron activation: key mechanisms in electroacupuncture's effect on pain-anxiety comorbidity
Source: Chin Med. 2025 Sep 2;20:143. doi: 10.1186/s13020-025-01202-1 (PMC12403460; doi:10.1186/s13020-025-01202-1)
Supplement: Supplementary file 1 — Additional file 1. [file 13020_2025_1202_MOESM1_ESM.docx]

**
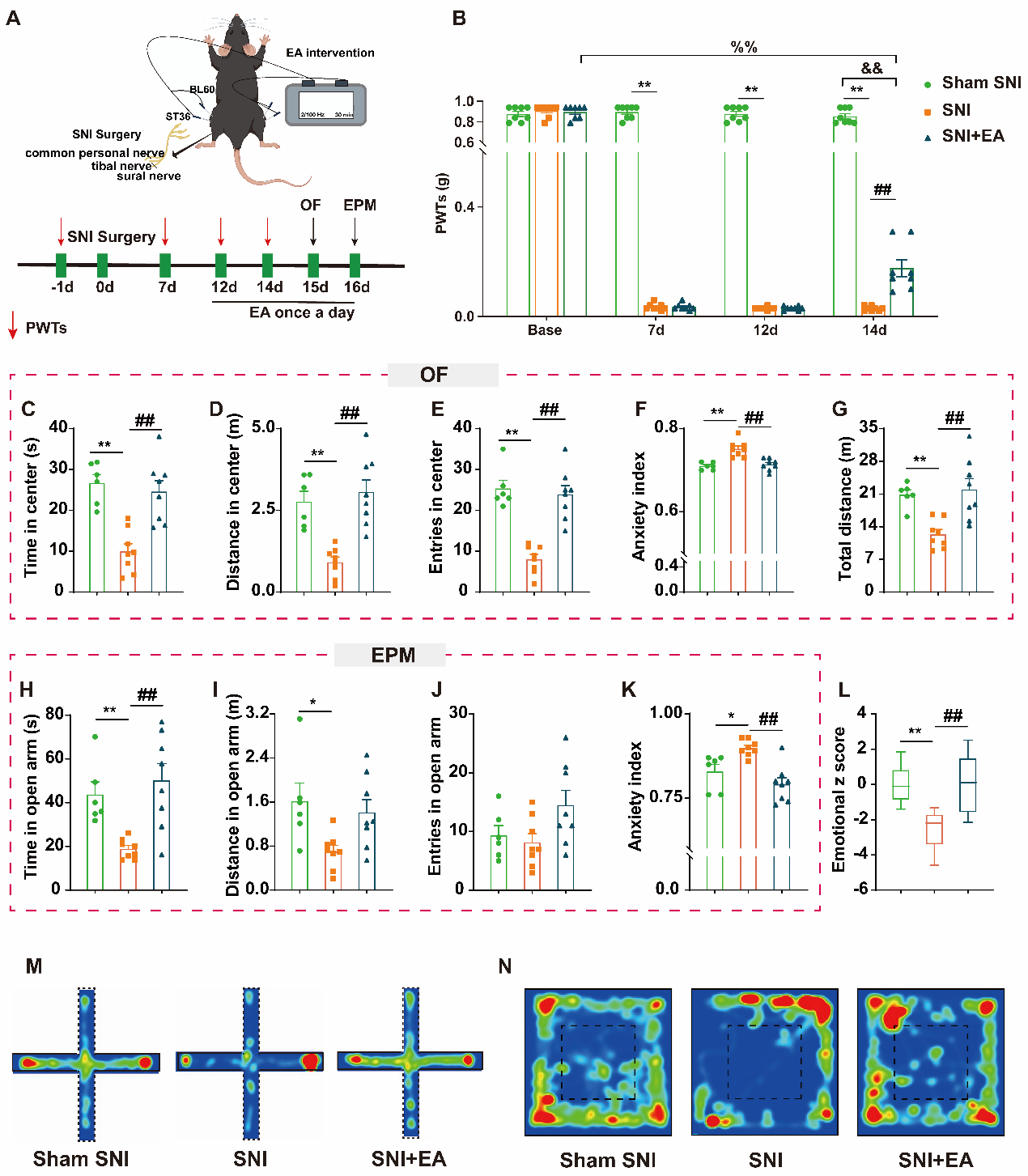
**

**Figure S1 EA attenuates pain and anxiety-like behaviors of SNI mice.**

A SNI model, EA intervention, and flow chart of experiments. B The effect of EA on PWTs (F_2,84_=1410, two-way ANOVA with Tukey’s multiple comparisons test, *P*<0.0001; n=8/group). C-G Result of OF. C Time in center of OF (F_2,19_=15.97, *P*<0.0001). D Distance in center of OF (F_2,19_=16.29, *P*<0.0001). E Entries in center of OF (F_2,19_=27.91, *P*<0.0001). F Anxiety index of OF (F_2,19_=16.63, *P*<0.0001). G Total distance in OF (F_2,19_=10.24, P=0.0010). H-K Result of EPM. Time in open arm of EPM (F_2,19_=9.146, P=0.0017). I Distance in open arm of EPM (F_2,19_=4.567, P=0.0240). J Entries in open arm of EPM (F_2,19_=3.080, P=0.0694). K Anxiety index (F_2,19_=11.48, P=0.0005). L Emotional z score (F_2,41_=21.64, *P*<0.0001). M Representative locomotion trace in OF. N Representative exploration traces in EPM. ***P*<0.01, Sham SNI v.s. SNI; ##P<0.01, SNI v.s. SNI+EA; &&*P*<0.01, Sham SNI v.s. SNI+EA, %% *P*<0.01, base of SNI+EA v.s.14d of SNI+EA. n=6-8 mice/group. Data are presented as the mean ± SEM. One-way ANOVA (C-L) with Tukey’s multiple comparisons test.


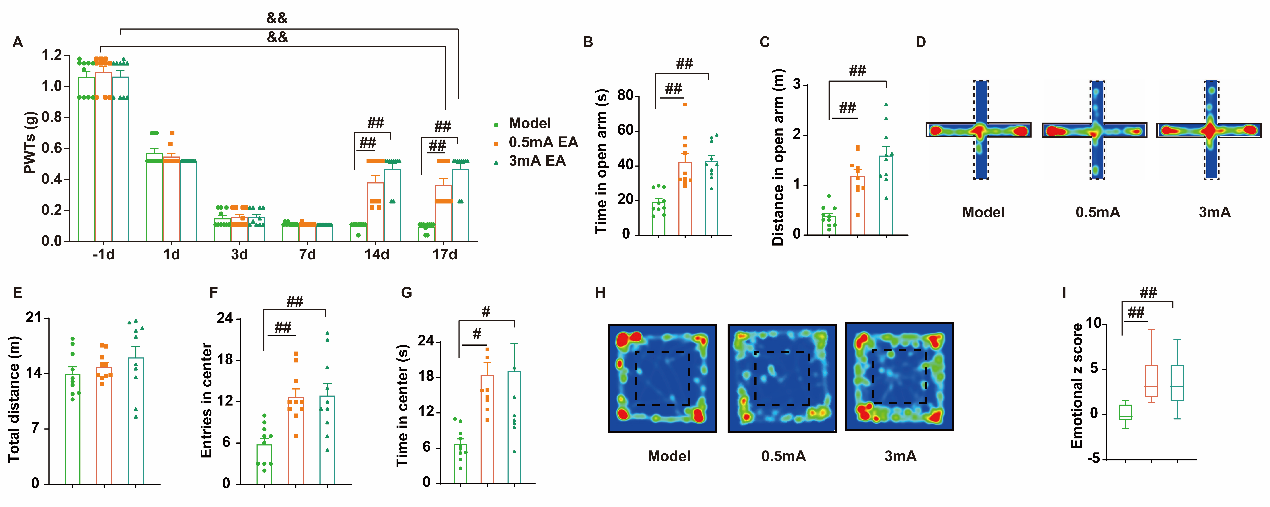


**Figure S2 Effect of different intensity EA on pain-anxiety comorbidity**

A The result of PWTs (F_2, 27_=17.01, two-way ANOVA with Tukey’s multiple comparisons test, P<0.0001; n=10/group). B-D Result of EPM. B Time in open arm of EPM (F_2,27_=14.21, P<0.0001). C Distance in open arm of EPM (F_2,27_=21.23, P<0.0001). D Representative exploration traces in EPM. E-H Result of OF, E Total distance of OF (F_2,27_=1.049, P=0.3640). F Entries in center of OF (F_2,27_=9.182, P=0.0009). G Time in center of OF (F_2,27_=5.341, P=0.0111). H Representative exploration traces of OF. I Emotional z score (F_2,57_=23.09, P<0.0001). # P<0.05, ## P<0.01, compared with Model group. && P<0.01, -1d v.s.17d. Data are presented as the means ± SEM. n=10 mice/group. One-way ANOVA (B-C, E-G, J-L, N-P, R) with Tukey’s multiple comparisons test.

**
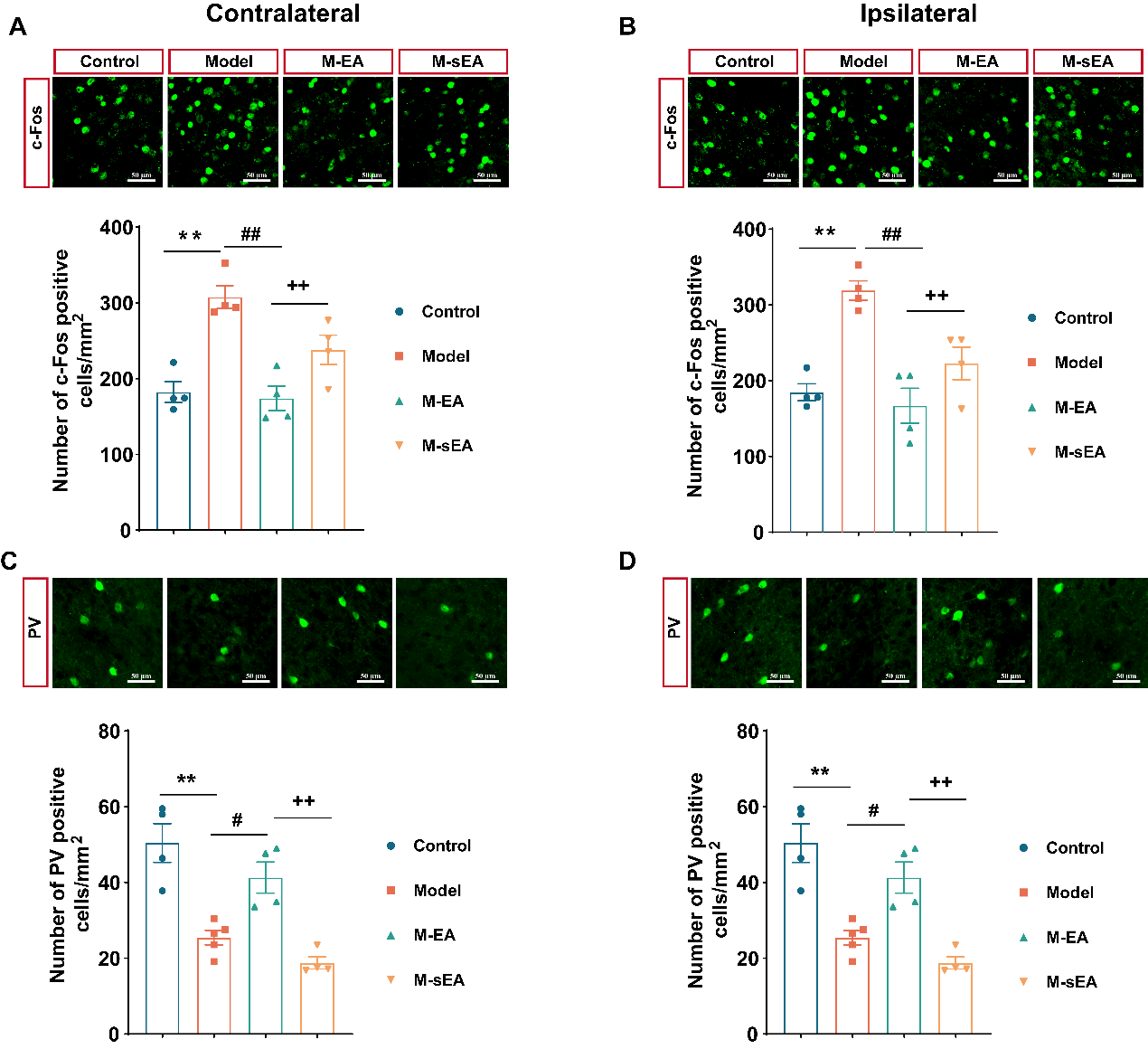
**

**Figure S3 EA downregulated the c-Fos expression and upregulated the PV positive cells in the ACC.**

A c-Fos expression levels in contralateral ACC (F_3, 12_=14.60, P=0.0003). B c-Fos expression levels in ipsilateral ACC (F_3, 12_=14.39, P=0.0003). C PV expression levels in contralateral ACC (F_3, 13_=18.04, P<0.0001). D PV expression levels in ipsilateral ACC (F_3, 13_=8.092, P=0.0027). ***P*<0.01, Control v.s. Model, #*P*<0.05, ##*P*<0.01, Model v.s. M-EA, ++P<0.01, M-EA v.s. M-sEA. Data are presented as the means ± SEM. n=3-5 mice/group.


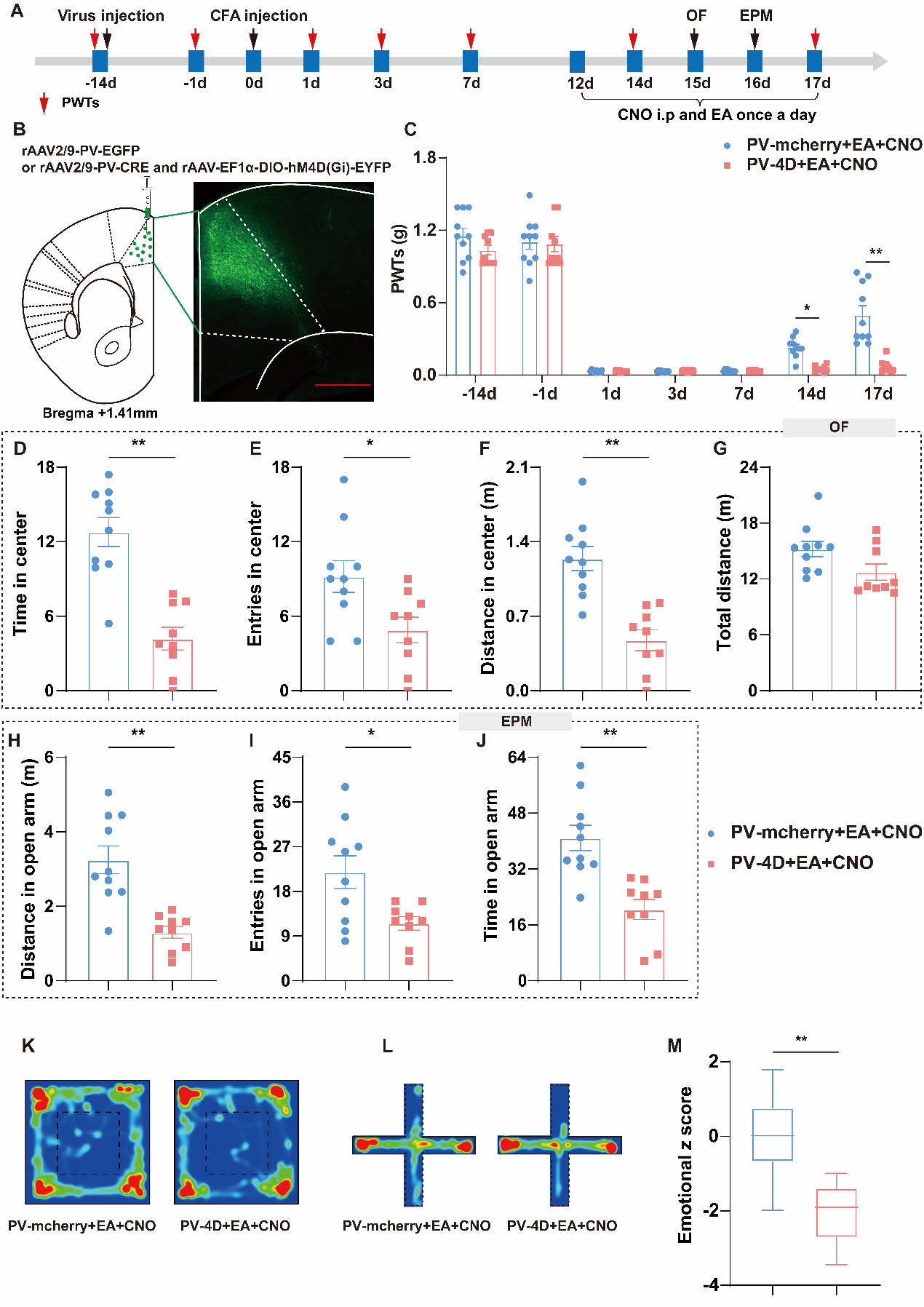


**Figure S4 Chemogenetic inhibiting of PV interneurons reverse the EA’s effect on relieving the pain-anxiety comorbidity.**

A Experimental scheme of virus injection, CFA injection, drug administration, EA intervention, and behavioral tests. B Virus injection strategy (left) and representative image show location of virus expression (right). C The results of PWTs (F_1,17_=33.16, P<0.0001). D-G Results of OF. D Time in center of OF (t=5.643, P<0.0001). E Entries in center of OF (t=2.594, P=0.0189). F Distance in center of OF (t=5.062, P<0.0001). G Total distance of OF (t=2.085, P=0.0525). H-J Results of EPM. H Distance in open arm of EPM (t=4.604, P=0.0003). I Entries in open arm of EPM (t=2.795, P=0.0124). J Time in open arm of EPM (t=4.333, P=0.0005). K Representative exploration traces of OF. L Representative exploration traces of EPM. M Emotional z score (t=7.092, P<0.0001). n=9-10 mice/group. Data are presented as the means ± SEM. Two-tailed unpaired *t*-test. **P*<0.05, ***P*<0.01, PV-mcherry+EA+CNO v.s. PV-4D+EA+CNO.


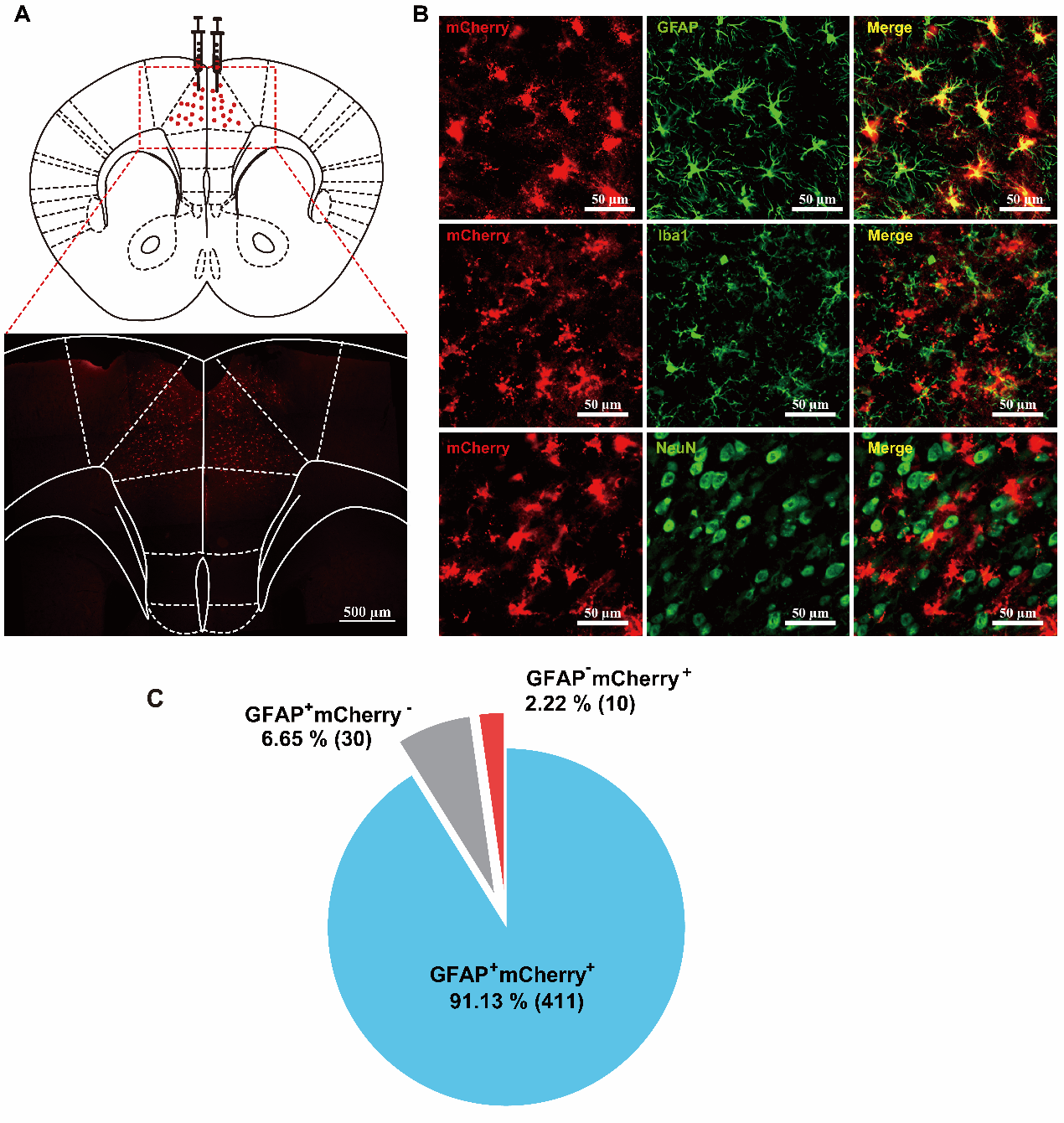


**Figure S5 Specificity of GFAP virus.**

A Schematic of bilateral virus infection. B Representative image of GFAP injection bites. C Quantitative result of coexpression of GFAP and mCherry.


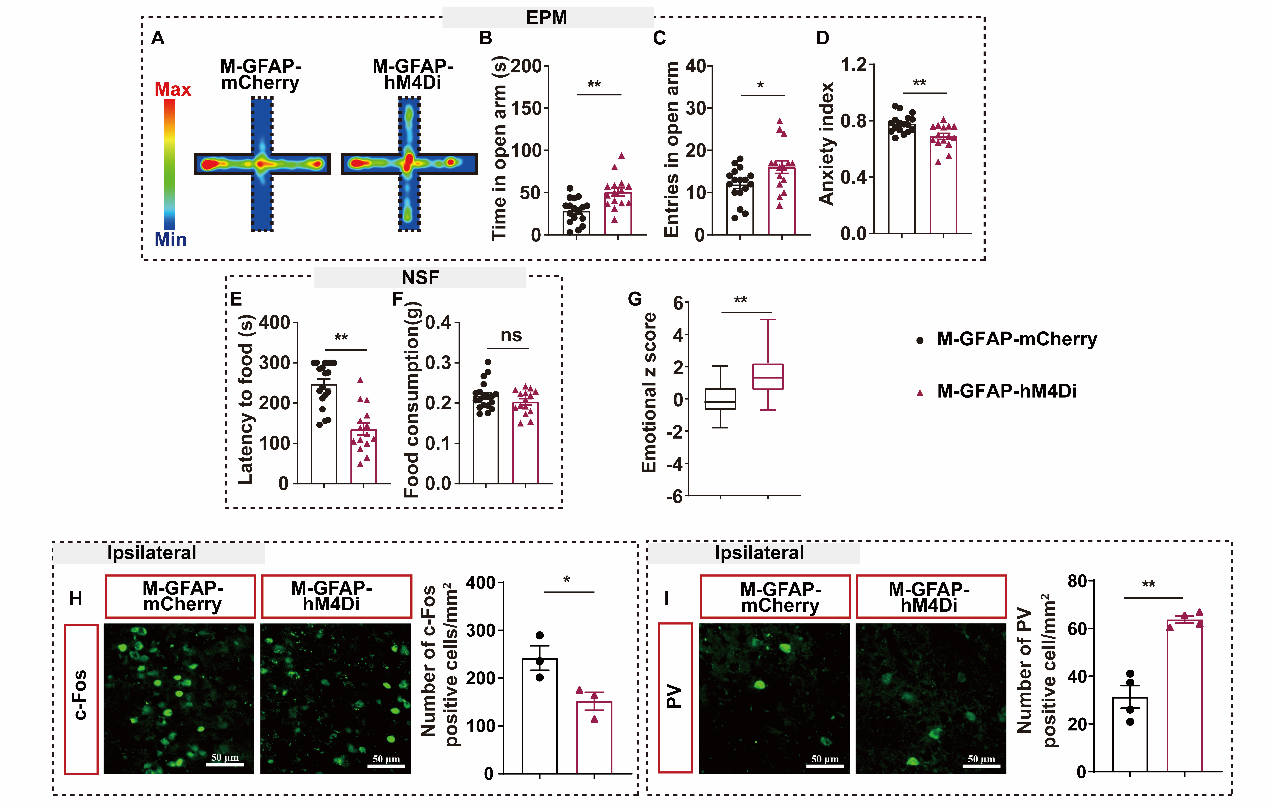


**Figure S6 The effect of GFAP on the pain-anxiety comorbidity.**

A-D Results of EPM. A Representative exploration traces in EPM. B Time in open arm of EPM (t=3.742, P=0.0008). C Entries in open arm of EPM (t=2.377, P=0.0240). D Anxiety index of EPM (t=3.427, P=0.0018). E-F Results of NSF. E Latency to food (t=5.775, P<0.0001). F Food consumption (t=1.360, P=0.1833). G Emotional z score (t=4.913, P<0.0001). Data are presented as the means ± SEM. n=12-15 mice/group. H Data representative for the c-Fos positive cells in the ipsilateral ACC (t=2.854, P=0.0462). I Data representative for PV positive cells in the ipsilateral ACC (t=6.546, P=0.0006). n=4 mice/group. **P*<0.05, ***P*<0.01, M-GFAP-mCherry v.s. M-GFAP-hM4Di; ns, no significant different (*P*>0.05). Two-tailed unpaired *t*-test.


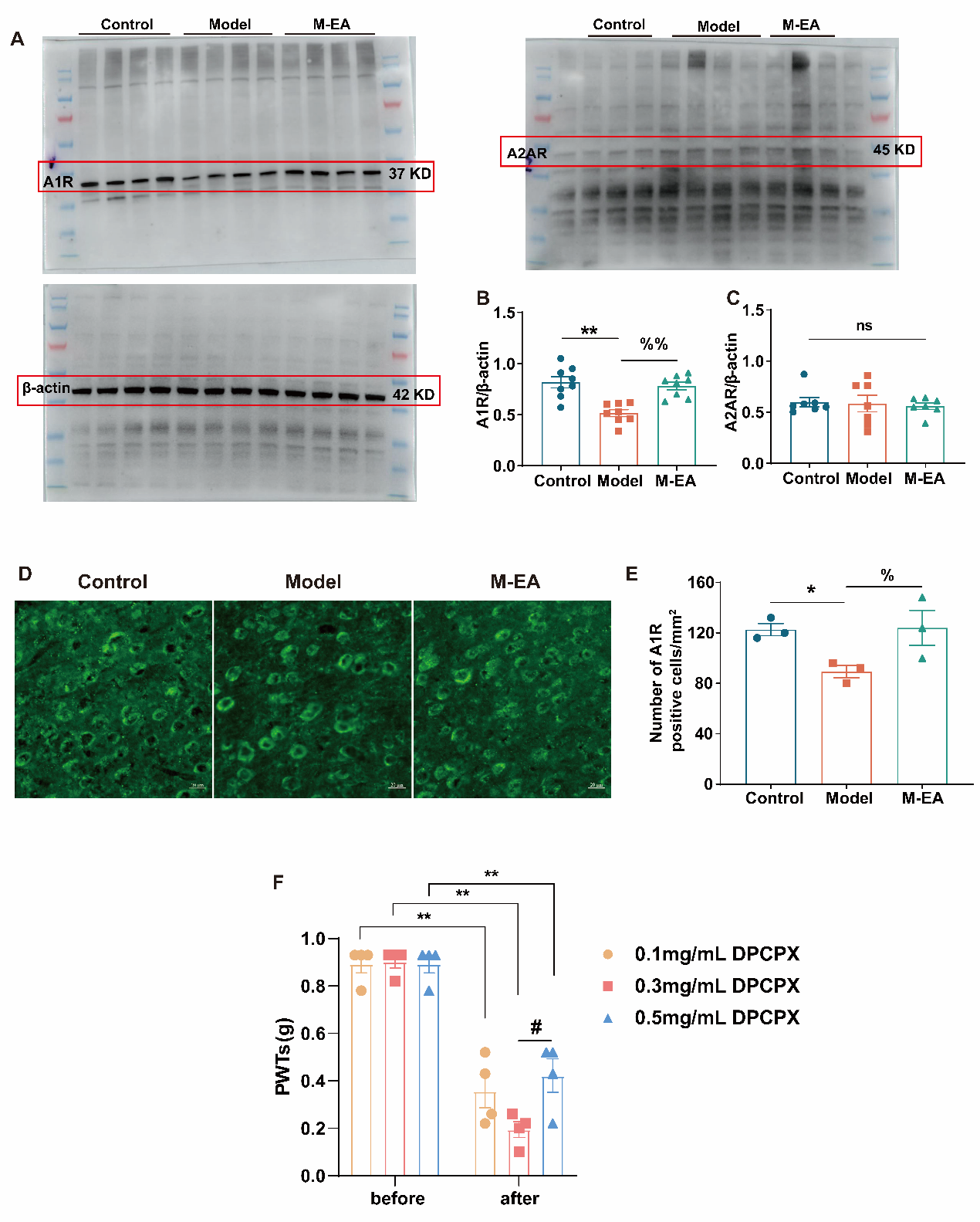


**Figure S7 The effect of EA intervention on A1R and A2AR in the ACC of pain-anxiety comorbidity mice**

A Representative band of WB, B Result of A1R protein expression in the ACC (F_2,21_=14.67, P=0.0001), C Result of A2AR protein expression in the ACC (F_2,18_=0.1170, P=0.8902). * *P*<0.05, ***P*<0.01, Control v.s. Model; % *P*<0.05, %% *P*<0.01. n=7-8/group. One-way ANOVA plus post hoc Tukey test. D Representative for the A1R positive cells in the ACC. E Result of A1R positive cells in the ACC (F_2,6_=8.326, P=0.0186). Bar=20 μm. n=3 mice/group. * *P*<0.05, Control v.s. Model; % *P*<0.05. F Effect of DPCPX at different concentrations on PWTs (F_2,9_=4.589, P=0.0423). ** *P*<0.05 before v.s. after; # *P*<0.05 0.3mg/mL DPCPX v.s. 0.5mg/mL DPCPX. n=4 mice/group. Data are presented as the means ± SEM. One-way ANOVA plus post hoc Tukey test.


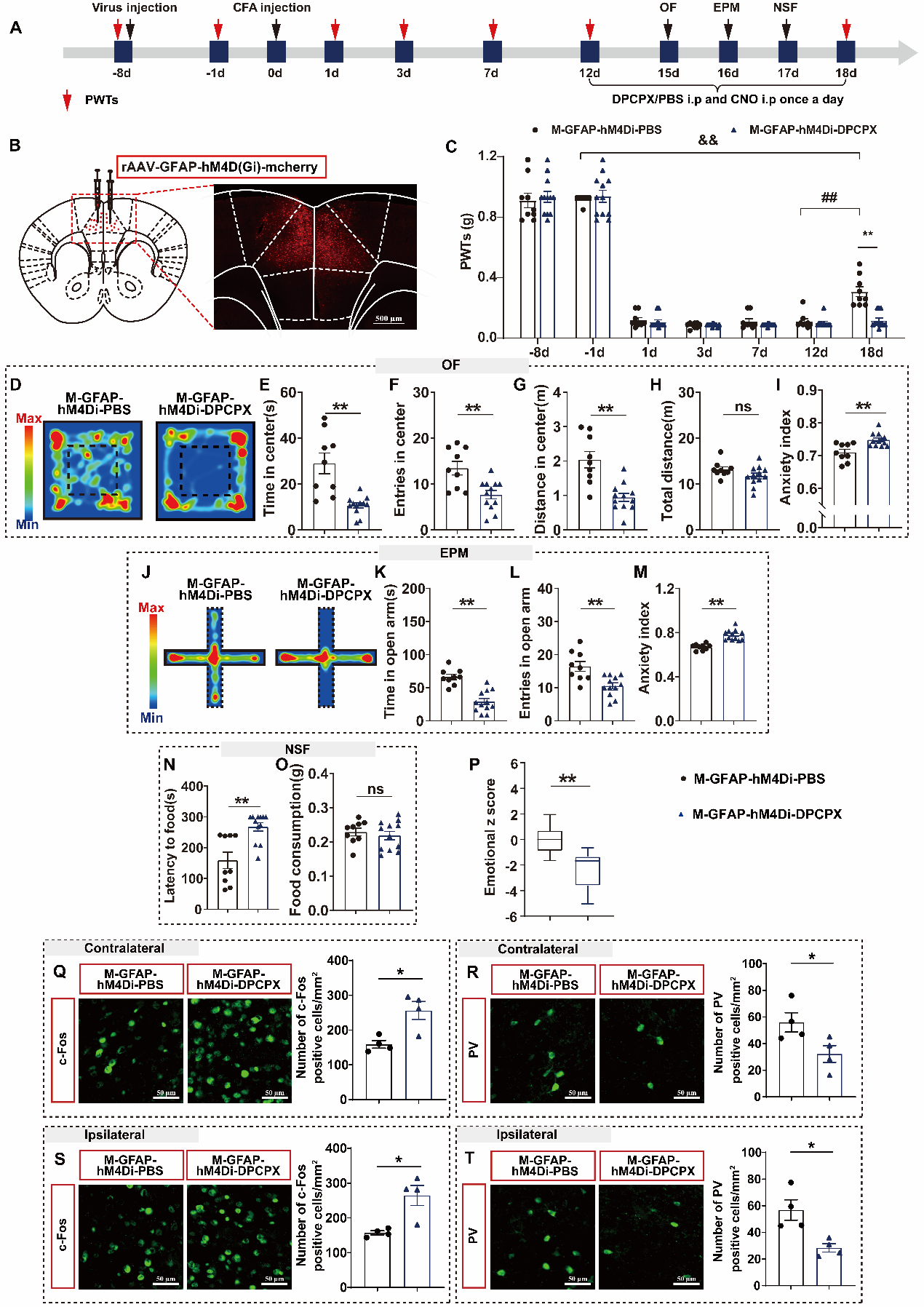


**Figure S8 The impact of intraperitoneal injection of A1R antagonist on the effect of GFAP inhibition on pain-anxiety comorbidity.**

A Experimental scheme of virus injection, CFA injection, drug administration, and behavioral tests. B Virus injection strategy (left) and representative image show location of virus expression (right). C The impact of DPCPX on the effect of GFAP inhibition on PWTs (on 18d, t=7.131, *P*<0.0001). D-I Results of OF. D Representative exploration traces of OF. E Time in center of OF (t=4.446, *P*=0.0003). F Entries in center of OF (t=3.420, *P*=0.0029). G Distance in center of OF (t=4.479, *P*=0.0003). H Total distance of OF (t=1.587, *P*=0.1289). I Anxiety index of OF (t=3.665, *P*=0.0016). J-M Result of EPM. J Representative exploration traces in EPM. K Time in open arm of EPM (t=5.755, *P*<0.0001). L Entries in open arm of EPM (t=3.558, *P*=0.0021). M Anxiety index of EPM (t=6.096, *P*<0.0001). N-O Result of NSF. N Latency to food (t=3.948, *P*=0.0009). O Food consumption (t=0.5938, *P*=0.5597). P Emotional z score (t=6.079, *P*<0.0001). n=9-12 mice/group. Q c-Fos positive cells in contralateral ACC (t=3.502, *P*=0.0128). R PV positive cells in contralateral ACC (t=2.48, *P*=0.0478). S Data representative for the c-Fos positive cells in ipsilateral ACC shown similarly to Q (t=3.626, *P*=0.011). T Data representative for PV positive cells in ipsilateral ACC shown similarly to R (t=3.420, *P*=0.0141). ***P*<0.01, M-GFAP-hM4Di-PBS v.s. M-GFAP-hM4Di-DPCPX; ns, no significant different (*P*>0.05). Data are presented as the means ± SEM. n=4 mice/group. Two-tailed unpaired *t*-test.


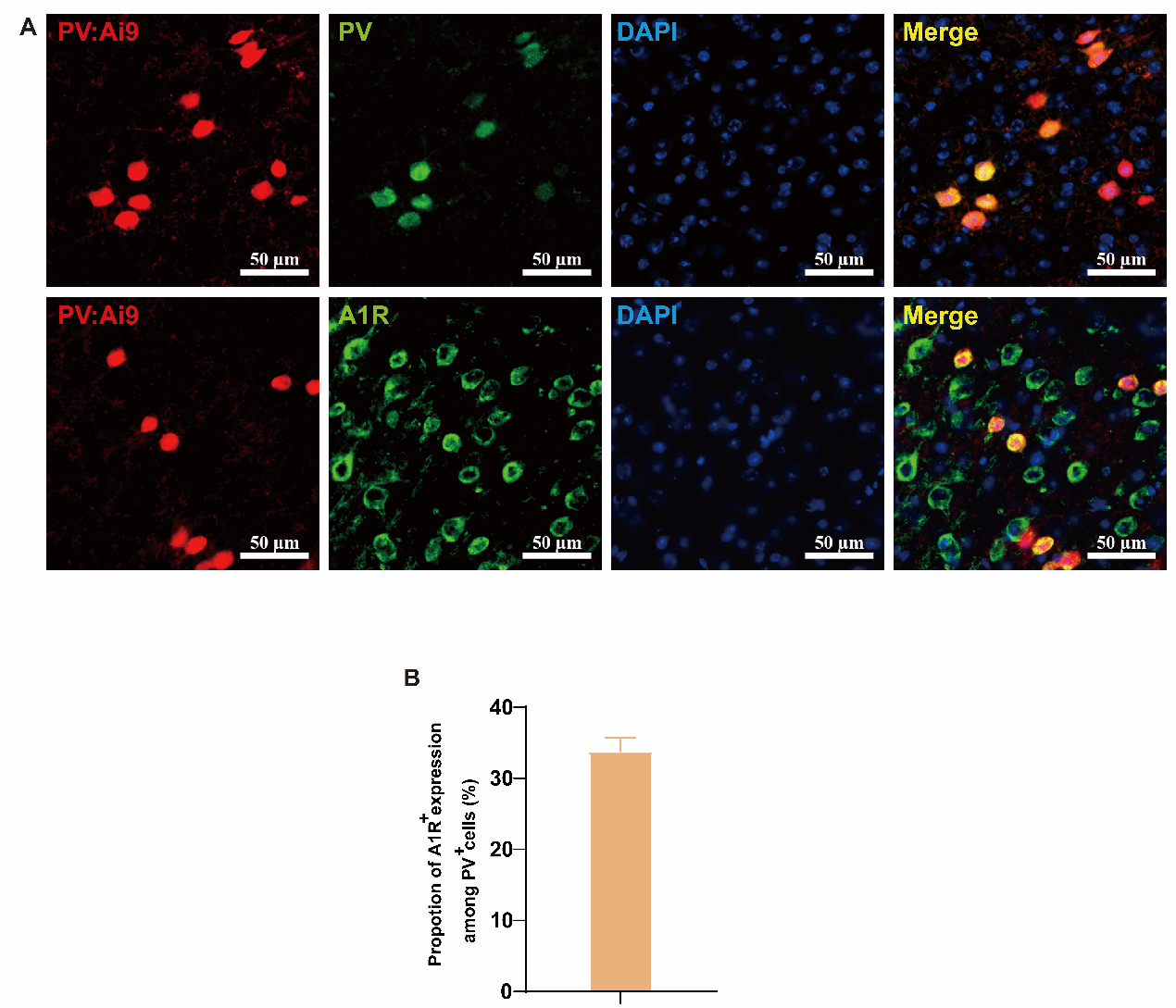


**Figure S9 Double immunofluorescences staining for PV and A1R.**

A A1R is primarily expressed in PV interneurons in the ACC, Brain slices from PV Cre:Ai9 mice were immunostained with antibodies against A1R (bottom, left second) and PV (up, left second). B Quantitative result of coexpression of A1R and PV positive cells.
